# Menthol carbonates as potent antiparasitic agents: synthesis and in vitro studies along with computer-aided approaches

**DOI:** 10.1186/s12906-022-03636-8

**Published:** 2022-06-13

**Authors:** Camila M. Clemente, Sara M. Robledo, Soledad Ravetti

**Affiliations:** 1 Instituto Multidisciplinario de Investigación y Transferencia Agroalimentaria y Biotecnológica (IMITAB), Instituto Académico Pedagógico de Ciencias Básicas y Aplicadas, Universidad Nacional de Villa María, Córdoba, Argentina; 2 Programa de Estudio y Control de Enfermedades Tropicales-PECET, Universidad de Antioquia, Medellín, Colombia; 3 Centro de Investigaciones y Transferencia Villa María (CIT VM), Instituto Académico Pedagógico de Ciencias Humanas, Universidad Nacional de Villa María, Arturo Jauretche 1555, X5900 Villa María, Córdoba, Argentina

**Keywords:** Menthol, Prodrugs, Cytotoxicity, Antiparasitic, Molecular dynamics

## Abstract

**Introduction:**

Despite the number of deaths and the significant economic and social costs associated with Chagas, Leishmaniasis and Malaria diseases worldwide, available drugs are limited and have serious side effects and high toxicity for the patient. Therefore, there is an urgent need for safe, low-cost, and effective treatments. Natural products are an important source of bioactive compounds and there is current interest in finding natural bioactive molecules that can be used for treating these parasitic diseases. In the present study we proposed to evaluate the in vitro antiparasitic activity of new menthol derivatives against *Trypanosoma cruzi*, *Leishmania braziliensis* and *Plasmodium falciparum;* moreover, we propose to explore their mode of action through in silico approaches.

**Material and methods:**

A series of carbonate prodrugs (**1**–**9**) were synthesized from menthol with different aliphatic alcohols. Spectroscopic techniques were used to confirm the structures of the synthesized compounds. The cytotoxicity of the compounds was assessed using U-937 cells. In vitro trypanocidal, leishmanicidal and antiplasmodial activity were evaluated using a *T. cruzi*, *L. braziliensis* and *P. falciparum* organism, respectively. In addition, in silico studies were also performed through molecular dynamics simulations and MM-PBSA analysis.

**Results:**

The assay revealed that most of the compounds were highly active against intracellular amastigotes of *T. cruzi* and *L. braziliensis*, and had moderate activity against the total forms of *P. falciparum*. Compound **2** was one of the drugs that showed a high selectivity index (SI) for the three organisms evaluated. The prediction of the ADME properties suggests that all the compounds have drug-like molecular properties and the probability to be lead candidates. Finally, molecular dynamics simulations, and MM-PBSA studies indicate that menthol at the substrate binding site of TcDHODH, LbDHODH and PfDHODH is structurally stable in the same order as the natural substrate; also, interactions of menthol with residues involved in the inhibition of TcDHODH and PfDHODH proteins were predicted.

**Conclusions:**

The present study demonstrates that menthol prodrugs are promising antiparasitic agents; however, the mechanisms of action proposed in this study need to be experimentally verified by future enzymatic assays.

**Supplementary information:**

The online version contains supplementary material available at 10.1186/s12906-022-03636-8.

## Introduction

Chagas disease, Leishmaniasis and Malaria are parasitic diseases that have common risk factors for transmission and disease development, such as poor housing, malnutrition, population mobility, lack of access to health services, and a weak immune system. These diseases are a major global health problem affecting more than one billion people worldwide, especially in low-income countries. In addition, population movements are currently spreading these diseases to other countries [1]. The current pharmacological treatment of these three parasitic infections has many disadvantages such as high toxicity, resistance, low efficacy in different life cycle stages of the parasites, and numerous side effects [2–5]. For this reason, the discovery of effective, safer, and inexpensive drugs is necessary.

Plants have been used for therapeutic purposes for centuries. Essential oils are complex aromatic mixtures of volatile compounds extracted from different parts of plants, such as flowers, buds, seeds, leaves, and others. 90% of the compounds in essential oils are monoterpenes [6]: Kolossa emphasizes that the chemistry, pharmacodynamics, and pharmacokinetics of terpenic compounds are different and explains that menthol differs from other components of terpenic essential oils [7].

Menthol (2-isopropyl-5-methylcyclohexano) is a cyclic alcohol monoterpene found as a major component in the essential oils of *Mentha canadensis L.* and *M. x piperita L.*, among others. Extracts of these essential oils are used in traditional medicine to treat various conditions, including infections, and also as insect repellent. Currently, several in vitro and in vivo studies have reported different biological properties of menthol such as analgesic, antibacterial, antifungal, anesthetic, and penetration-enhancing activity, as well as its chemopreventive and immunomodulatory actions [8, 9].

Recent studies demonstrated the high potential of menthol as an antiparasitic agent. Zaia et al. (2016) found that a formulation composed of menthol (30–55%) and menthone (14–32%) was able to decrease the number of *Schistosoma mansoni* eggs in feces, liver, and intestine of infected mice but also reduced the number of hepatic granulomas; a reduction of eosinophilia in blood and a decrease of IL-4 and IL-10 levels in the blood after treatment were also observed [10]. Subsequently, Feitosa et al. (2018) found that a combination of menthol and menthone associated with acetylsalicylic acid reduces the hepatic inflammation and fibrosis in the murine schistosomiasis model [11]. Furthermore, Mohammad et al. (2020) reported that the *M. pulegium* L. extract composed of menthol (99.23%) and limonene (0.227%) has low cytotoxicity and high activity against the *Toxoplasma gondii* [12]. Additionally, Nikitin et al. (2021) reported in vitro activity of two dithiophosphoric derivatives of menthol against for *Caenorhabditis elegans* with death of 89.5–98.5% of the parasites after 24 h of exposure at a concentration of 2000 μg/mL [13].

Fabbri et al. (2020) reported that the menthol associated with pentanol showed a greater protoscolicidal effect than menthol in *Echinococcus multilocularis* protoscoleces but also a clinical efficacy similar to Albendazole. Thus, defining the menthol-pentanol prodrug as a promising candidate for its potential alternative for the treatment of alveolar echinococcosis [14].

Despite the diverse biological activities attributed to menthol, it exhibits poor physicochemical stability, limited aqueous solubility, and poor bioavailability which hamper its therapeutic use in medicine [15].

Continuing our studies on the development of antiparasitic agents using the pharmaceutical strategy of prodrugs [14, 16, 17] and taking into account what has been described above, we report the synthesis and in vitro antiprotozoal evaluation of new menthol carbonates against *Trypanosoma cruzi*, *Leishmania braziliensis* and *Plasmodium falciparum*.

Besides, a theoretical computational ADME study of the compounds and an in silico analysis of molecular dynamics simulations were performed to investigate a possible interaction of menthol at the substrate-binding site of the enzymes dihydroorotate dehydrogenases from *T. cruzi* (TcDHODH), *L. braziliensis* (LbDHODH), and *P. falciparum* (PfDHODH).

## Material and methods

### Chemistry

All chemicals, reagents, and solvents were of analytical grade. 2-Isopropyl-5-methylcyclohexanol (menthol purest ≥ 99%, Sigma-Aldrich) and 1,1-carbonyldiimidazole (CDI, purest ≥ 97%, Sigma-Aldrich) were used without purification. Dichloromethane (CH_2_Cl_2_) was distilled and dried over 4 Å molecular sieves. All solid reagents were dried for several hours under a high vacuum. TLC was performed on Merck Sil G/UV254 silica gel plates with fluorescent indicators. The chromatoplates were observed with UV light at 254 nm. All glassware was oven-dried at 130 °C overnight and cooled in a desiccator over anhydrous CaSO_4_ [16, 17]. The ^1^H and ^13^C nuclear magnetic resonance (NMR) spectra were recorded on a 400 MHz Brüker Advance II spectrometer. Chemical shift values are reported in d units relative to tetramethylsilane as internal standard and coupling constants (J) are given in hertz (Hz). The splitting pattern abbreviations are as follows: s for singlet signal, d for doublet signal, t for triplet signal, and m for multiple signal. All ^13^C-NMR spectra were proton decoupled. The structure of each compound was elucidated by a combined analysis of Fourier transform infrared spectroscopy (FTIR). The clear formation of carbonate and methylation occurred in all cases. High-resolution accurate mass (HRMS) measurements were performed using a micrOTOF QII quadrupole time-of-flight mass spectrometer (QTOF; Bruker Daltonics). The individual data are described below:

- 2-Isopropyl-5-methylcyclohexanol (Menthol). ^1^H-NMR (400 MHz, CHCl_3_) (ppm): 3.40 (m, 1H, H-1), 2.6 (m, 1H, H-7), 1.97–1.10 (m, 9H, H-2, H-3, H-4, H-5, H-6, H-7), 0.85–0.80 (m, 9H, H-8, H-9, H-10), ^13^C-﻿NMR (101 MHz, CHCl_3_) (ppm): 71.6 (C-1), 50.2 (C-2), 45.1 (C-6), 34.5 (C-4), 31.6 (C-5), 25.8 (C-7), 23.2 (C-3), 22.2 (C-10), 21.0 (C-9), 16.1 (C-8), IR-FTIR (cm-1): 3245.3 (OH), HRMS m/z calculated for C_10_H_20_O [M-H] + 156.1514, found: 156.2655.
- Metyl(2-Isopropyl-5-methylcyclohexanol)carbonate (**1**). ^1^H-﻿NMR (400 MHz, CHCl_3_) (ppm): 4.85 (m, 1H, H-1), 4.13 (s, 3H, H-1’), 2.13–1.20 (m, 9H, H-2, H-3, H-4, H-5, H-6, H-7), 0.91–0.76 (m, 9H, H-8, H-9, H-10), ^13^C-﻿NMR (101 MHz, CHCl_3_) (ppm): 148.8 (C-11), 91.4 (C-1), 54.6 (C-1’), 47.0 (C-2), 44.4 (C-6), 33.6 (C-4), 31.6 (C-5), 26.5 (C-7), 23.5 (C-3), 21.7 (C-10), 20.9 (C-9), 16.3 (C-8), IR-FTIR (cm-1): 1753.0 (OC(O)O), HRMS m/z calculated for C_12_H_22_O_3_ [M-H] + 215.1568, found: 217.1424.
- Etyl(2-Isopropyl-5-methylcyclohexanol)carbonate (**2**). ^1^H-﻿NMR (400 MHz, CHCl_3_) (ppm): 4.81 (m, 1H, H-1), 4.41 (m, 2H, H-1’), 2.09–1.07 (m, 12H, H-2, H-3, H-4, H-5, H-6, H-7, H-2’), 0.87–0.72 (m, 9H, H-8, H-9, H-10), ^13^C-﻿NMR (101 MHz, CHCl_3_) (ppm): 148.6 (C-11), 79.3 (C-1), 64.3 (C-1’), 47.0 (C-2), 40.5 (C-6), 33.9 (C-4), 31.3 (C-5), 26.5 (C-7), 23.5 (C-3), 21.8 (C-10), 16.4 (C-8, C-9), 14.0 (C-2’), IR-FTIR (cm-1): 1752.00 (OC(O)O), HRMS m/z calculated for C_13_H_24_O_3_ [M-Na] + 250.1725, found: 251.1375.
- Propyl(2-Isopropyl-5-methylcyclohexanol)carbonate (**3**). ^1^H-﻿NMR (400 MHz, CHCl_3_) (ppm) 4.86 (m, 1H, H-1), 3.57 (m, 2H, H-1’), 2.14–1.12 (m, 11H, H-2, H-3, H-4, H-5, H-6, H-7, H-2’), 0.90–0.77 (m, 12H, H-8, H-9, H-10, H-3’), ^13^C-﻿NMR (101 MHz, CHCl_3_) (ppm): 148.3 (C-11), 79.5 (C-1), 69.2 (C-1’), 47.0 (C-2), 40.5 (C-6), 33.9 (C-4), 31.4 (C-5), 26.5 (C-7), 23.5 (C-3), 21.8 (C-10), 20.6 (C-2’), 16.4 (C-8, C-9), 10.2 (C-3’), IR-FTIR (cm-1): 1752.00 (OC(O)O), HRMS m/z calculated for C_14_H_26_O_3_ [M-H] + 243.1881, found: 243.3623.
- Isopropyl(2-Isopropyl-5-methylcyclohexanol)carbonate (**4**). ^1^H-﻿NMR (400 MHz, CHCl_3_) (ppm): 5.26 (m, 1H, H-1), 4.87 (m, 1H, H-1’), 1.89–1.50 (m, 15H, H-2, H-3, H-4, H-5, H-6, H-7, H-2’, H-3’), 0.93–0.78 (m, 9H, H-8, H-9, H-10), ^13^C-﻿NMR (101 MHz, CHCl_3_) (ppm): 148.3 (C-11), 73.2 (C-1. C-1’), 47.0 (C-2), 40.5 (C-6), 33.9 (C-4), 31.4 (C-5), 26.5 (C-7), 23.5 (C-3), 21.8 (C-10), 20.6 (C-2. C-3’), 16.4 (C-8, C-9), IR-FTIR (cm-1): 1752.00 (OC(O)O), HRMS m/z calculated for C_14_H_26_O_3_ [M-H] + 242.1881, found: 242.1954.
- Butyl(2-Isopropyl-5-methylcyclohexanol)carbonate (**5**). ^1^H-﻿NMR (400 MHz, CHCl_3_) (ppm): 4.82 (m, 1H, H-1), 3.33 (m, 2H, H-1’), 2.11–1.18 (m, 13H, H-2, H-3, H-4, H-5, H-6, H-7, H-2’, H-3’), 0.87–0.74 (m, 12H, H-8, H-9, H-10, H-3’), ^13^C-﻿NMR (101 MHz, CHCl_3_) (ppm): 148.2 (C-11), 79.4 (C-1), 71.0 (C-1’), 47.0 (C-2), 40.5 (C-6), 33.9 (C-4), 31.4 (C-5), 29.7 (C-2’), 26.5 (C-7), 23.5 (C-3), 21.8 (C-10), 20.6 (C-8, C-9, C-3’), 16.4 (C-4’), IR-FTIR: 1743.56 (OC(O)O), HRMS m/z calculated for C_16_H_28_O_3_ [M-H] + 257.2038, found: 257.3889.
- Pentyl(2-Isopropyl-5-methylcyclohexanol)carbonate (**6**). ^1^H-﻿NMR (400 MHz, CHCl_3_) (ppm): 4.43 (m, 1H, H-1), 4.03 (m, 2H, H-1’), 2.14–1.25 (m, 15H, H-2, H-3, H-4, H-5, H-6, H-7, H-2’, H-’3, H-4’), 0.82–0.73 (m, 12H, H-8, H-9, H-10, H-5’), ^13^C-﻿NMR (101 MHz, CHCl_3_) (ppm): 155.1 (C-11), 78.2 (C-1), 71.0 C-1’), 47.0 (C-2), 40.5 (C-6), 33.9 (C-4), 31.4 (C-5), 29.7 (C-2’), 26.5 (C-7), 23.5 (C-3), 21.8 (C-10), 20.6 (C-8, C-9, C-3’), 16.4 (C-4’), 14.0 (C-5’), IR-FTIR (cm-1): 1742.1 (OC(O)O), HRMS m/z calculated for C_16_H_30_O_3_ [M-H] + 271.2194, found: 271.4156. This compound was previously characterized by Fabbri et al. [12].
- Hexyl(2-Isopropyl-5-methylcyclohexanol)carbonate (**7**). ^1^H-﻿NMR (400 MHz, CHCl_3_) (ppm): 5.18 (m, 1H, H-1), 3.52 (m, 2H, H-1’), 2.07–1.19 (m, 17H, H-2, H-3, H-4, H-5, H-6, H-7, H-2’, H-’3, H-4’, H-5’), 0.85–0.70 (m, 12H, H-8,H-9, H-10, H-6’), ^13^C-﻿NMR (101 MHz, CHCl_3_) (ppm): 148.5 (C-11), 79.4 (C-1), 68.8 (C-1’), 46.9 (C-2), 40.5 (C-6), 33.8 (C-4), 31.5 (C-4’), 31.3 (C-5), 28.2 (C-2’), 25.5 (C-7), 25.2 (C-3’), 23.4 (C-3), 22.5 (C-5’), 21.7 (C-10), 20.4 (C-8, C-9), 16.3 (C-6’), IR-FTIR (cm-1): 1743.5 (OC(O)O), HRMS m/z calculated for C_17_H_32_O_3_ [M-Na] + 307.2351, found: 307.2638.
- Heptyl(2-Isopropyl-5-methylcyclohexanol)carbonate (**8**). ^1^H-﻿NMR (400 MHz, CHCl_3_) (ppm): 4.41 (m, 1H, H-1), 3.61 (t, J = 6.7 Hz, 2H, H-1’), 1.56–1.28 (m, 19H, H-2, H-3, H-4, H-5, H-6, H-7, H-2’, H-’3, H-4’, H-5’, H-6’), 0.96–0.81 (m, 12H, H-8, H-9, H-10, H-7’), ^13^C-﻿NMR (101 MHz, CHCl_3_) (ppm): 148.6 (C-11), 79.4 (C-1), 68.5 (C-1’), 47.0 (C-2), 40.5 (C-6), 34.0 (C-4), 32.7 (C-5’), 31.5 (C-5), 29.0 (C-2’), 28.8 (C-4’), 26.5 (C-3’), 25.7 (C-7), 23.5 (C-3), 22.5 (C-6’), 21.8 (C-10), 20.5 (C-8, C-9), 16.3 (C-6’), 14.0 (C-7’), IR-FTIR (cm-1): 1758.0 (OC(O)O), HRMS m/z calculated for C18H34O3 [M-Na] + 320.9507, found: 321.2341.
- Octyl(2-Isopropyl-5-methylcyclohexanol)carbonate (**9**), ^1^H-﻿NMR (400 MHz, CHCl_3_) (ppm) 4.76 (m, 1H, H-1), 4.27 (m, 2H, H-1’), 2.04–1.14 (m, 21H, H-2, H-3, H-4, H-5, H-6, H-7, H-2’, H-’3, H-4’, H-5’, H-6’, H-7’), 0.80–0.67 (m, 12H, H-8, H-9, H-10, H-8’), ^13^C-﻿NMR ﻿(101 MHz, CHCl_3_) (ppm): 155.3 (C-11), 79.3 (C-1), 68.4 C-1’), 46.9 (C-2), 40.4 (C-6), 33.8 (C-4), 32.7 (C-6’), 31.4 (C-5), 28.9 (C-4’), 28.3 (C-5’), 26.4 (C-3’), 25.7 (C-7), 23.4 (C-3), 22.4 (C-7’), 21.7 (C-10), 20.4 (C-8, C-9), 13.8 (C-8’), IR-FTIR (cm-1): 1743.0 (OC(O)O), HRMS m/z calculated for C_19_H_36_O_3_ [M-Na] + 335.2664, found: 335.2549.

#### General procedure for the synthesis of carbonates of menthol.

One equiv of menthol (200 mg, 1.2 mmol) under the N_2_ stream was added to 1.2 equiv of 1,1-carbonyldiimidazole (CDI; 233 mg; 1.44 mmol) in dried dichloromethane (CH_2_Cl_2_, 10 mL). The mixture reaction was stirred at room temperature for 2 h leading to an intermediate of menthol (menthol-1-carbonylimidazole). The progress of the reaction was monitored by thin-layer chromatography (TLC) n-hexane/AcOEt, 6:4 v/v. Once, aliphatic alcohol (1.5 equiv) was added, and the reaction mixture was maintained in the same conditions until total conversion of menthol-1-carbonylimidazole with the formation of the corresponding carbonate product. The organic phase was successively washed with water (3 × 20 mL). The organic layer was dried over Na_2_SO_4_, filtered, and the solvent was evaporated under reduced pressure [16, 17].

### Biological activity assays

All compounds (menthol, **1**–**9**) were subjected to in vitro evaluation of their cytotoxicity against U-937 human macrophages and leishmanicidal, trypanocidal, and antiplasmodial activities in intracellular amastigotes of *L. braziliensis*, *T. cruzi*, and *P. falciparum*, respectively. Compounds were solubilized in DMSO (1 mg/mL, w/v). Stock solutions at 200 g/mL (v/v) in complete RPMI-1640 medium were prepared and then four-fold serial dilutions of each compound were prepared. At the end, each compound was tested at different concentrations (200, 50, 12.5, 3.125 and 0.78 µg/mL) [16, 17].

#### In vitro cytotoxicity

Human macrophages of the U-937 cell line were maintained in culture in RPMI-1640 medium enriched with 10% fetal bovine serum (FBS) and 1% penicillin–streptomycin solution. The cells were adjusted at 1 × 10^5^ cells/mL in enriched RPMI–1640. Then, 100 µL of cell suspension and 100 μL of each concentration of the compounds were placed in each well of 96-well tissue culture microplate, and plates were incubated again at 37 °C, 5% CO_2_. After 72 h of incubation 20 μL (0.5 mg/mL) of MTT dissolved in serum-free RPMI-1640 medium were added to each well and plates were incubated for 3 h at 37 °C, 5% CO_2_. The reaction was stopped with 100 μL/well of DMSO and absorbance was recorded at 570 nm (Varioskan Flash, Thermo Scientific, Waltham, MA, USA). Doxorubicin (DOXO) was used as positive control and enriched RPMI-1640 medium was used as a negative control. A blank solution (enriched RPMI-1640 medium plus 0.2% DMSO) was used to correct the absorbance. Each measurement was done in triplicate in two independent experiments [16, 17].

#### In vitro activity for *T.cruzi* amastigotes

The U-937 cells were adjusted at 2.5 × 10^6^ cells/ml in enriched RPMI–1640 plus 0.1 µg/mL phorbol myristate acetate (PMA) and 100 µL were placed in each well of 96-well tissue culture microplate. Plates were incubated at 37 °C, 5% CO_2_. After 72 h of incubation, cells were infected with 5:1 epimastigotes of *Tulahuen* strain (24 h of growing) per cell ratio. Plates were incubated at 37 °C, 5% CO_2_. After 24 h of incubation 100 μL of each compound concentration were added to each well and plates were incubated again at 37 °C, 5% CO_2_. After 72 h of incubation 100 μL of chlorophenol red-β-D-galactopyranoside (CPRG) at 100 µM and 0.1%, Nonidet P-40 was added, and after 3 h of incubation, absorbance was read at 570 nm (Varioskan, Thermo) [18]. Infected cells exposed to Benznidazole (BZN) were used as a control for trypanocidal activity (positive control) while infected and non-treated cells were used as a control for infection (negative control). Nonspecific absorbance was corrected as described above. Each measurement was done in triplicate in two independent experiments [16, 17].

#### In vitro activity against *L. braziliensis* amastigotes

The U-937 cells were adjusted at 10^5^ cells/mL of enriched RPMI-1640 plus 0.1 µg/mL PMA. One ml was dispensed into each well of a 24-well culture plate. After 72 h, cells were infected with promastigotes of *L. braziliensis* (HMOM/COL/88/UA301-EGFP) in a proportion of 15:1 (parasites per cell ratio). Plates were incubated at 34 °C, 5% CO_2_, and 3 h after non-internalized promastigotes were removed by washing twice with phosphate buffer (PBS). Plates were incubated again at 34 °C, 5% CO_2_, and 24 h later cells were washed with warm PBS and medium replaced. One-hundred μl/well of each compound dilution (having as a starting point twice the LC_50_ of the corresponding compound) were added to each well and plates were incubated at 37 °C with 5% CO_2_. After 72 h, cells were removed using trypsin/EDTA solution and washed twice with PBS by centrifuging 10 min at 1100 rpm, 4 °C. Cells were read by flow cytometry at 488 nm excitation and counting 10.000 events. The percentage of infected cells was calculated using dot plot analysis according to positive events for green fluorescence and then parasite load in these infected cells was calculated by histogram analysis according to the mean fluorescence intensity (MFI). Infected cells were used as control of infection (negative control) and infected cells exposed to Amphotericin B (AMB) were used as control of antileishmanial activity (positive control) [19, 20].

#### In vitro antiplasmodial activity

Unsynchronized *P. falciparum *(3D7 strain) culture was adjusted to 0.5% parasitemia and 1% hematocrit in RPMI medium enriched with 3% lipid-rich bovine serum albumin-Albumax II. In each well of a 96-well tissue culture plate, 100 µl of parasite suspension and 100 µl of each dilution of the compounds were added. Plates were incubated for 48 h at 37 °C, 90% N_2_, 5% CO_2_, 5% O_2_. After incubation, supernatants were harvested and parasites were subjected to three 20–minute freeze–thaw cycles. In a second flat–bottomed 96-well microtiter plate were dispensed 100 µL of Malstat reagent, pH 9.0, 25 µL of NBT/PES solution, and 15 µL of lysed parasites. Plates were incubated in the dark at 37 °C for 90 min and color development of the pLDH reaction was read at 650 nm (Varioskan, Thermo) [21]. Chloroquine (CQ) was used as a positive antiplasmodial drug control. Parasites cultured in enriched RPMI-1640 were used as a control of both growth and viability (negative control). Nonspecific absorbance was corrected by subtracting optical density (OD) of the blank. Nonspecific absorbance was corrected as described above. Each measurement was done in triplicate in two independent experiments.

#### Data analysis

To calculate the cytotoxicity, the percentages of mortality for each concentration tested were calculated according to Eq. 1:

1 $$\%\mathrm{Mortality}=100-\left[\left(\mathrm{OD}\;\mathrm{exposed}\;\mathrm{cells}/\mathrm{OD}\;\mathrm{non}\;\mathrm{exposed}\;\mathrm{cells}\right)\mathrm x\;100\right]$$

Then, these percentages of mortality were used to calculate the median lethal concentrations (LC_50_) by Probit analysis using Graphpad Prism 8 software. In the case of antiparasitic activity (*L. braziliensis*, *T. cruzi* and *P. falciparum*), the percentage in the reduction of parasites for each concentration tested was quantified using Eq. 2:

2 $$\%\mathrm{Parasite}\;\mathrm{reduction}=100\;\left[\left(\mathrm{OD}\;\mathrm{or}\;\mathrm{MFI}\;\mathrm{exposed}\;\mathrm{parasites}/\;\mathrm{OD}\;\mathrm{or}\;\mathrm{MFI}\;\mathrm{control}\;\mathrm{parasites}\right)\;\mathrm x100\right]$$

The preference of the activity for each compound was determined according to the IS which corresponds to the ratio between cytotoxicity and antiparasitic activity and calculated as the quotient when dividing the LC_50_ and the EC50, Eq. 3:

3 $$\mathrm{IS}\;=\;{\mathrm{LC}}_{50}/\;{\mathrm{EC}}_{50}$$

All experiments were performed in triplicate. Both cytotoxicity and antiparasitic activity were classified as high, moderate, or low, according to ranges previously established based on the own hit criteria. High cytotoxicity: LC_50_ < 50 μM, moderate: LC_50_ > 50 μM < 100 μM) and low cytotoxicity: LC_50_ > 100 μM). Similarly, the anti-protozoal activity was classified grouped according to EC_50_ values, based on the own criteria into high (EC_50_ < 50 μM), moderate (EC_50_ > 50 μM < 100 μM) and low activity (EC_50_ > 100 μM). Compounds with IS of 3 or more were considered selective.

### Computational chemistry

#### Drug-likeness

The drug-likeness and ADME properties for menthol and its prodrugs were calculated using the SwissADME webserver [22].

#### Molecular docking and dynamics studies

All proteins were collected from the Protein Data Bank [23] (PDB, http://www.rcsb.org/). The proteins selected were *Trypanosoma cruzi* dihydroorotate dehydrogenase (TcDHODH, PDB: 2E68/Chain B), *Leishmania braziliensis* dihydroorotate dehydrogenase (LbDHODH, PDB: 4WZH/Chain B), and *Plasmodium falciparum* dihydroorotate dehydrogenase (PfDHODH, PDB: 4CQA/Chain B). All the molecules that are not part of the structure of the protein were removed. Hydrogen atoms were added to the proteins and converted to pdbqt format using the MGL tools of the AutoDockTools4 software [24] for later analysis. The 2D structures of the ligands were downloaded from PubChem (https://pubchem.ncbi.nlm.nih.gov) in SDF format and then converted to PDB format using the Open Babel [25] software. Hydrogen atoms were added to the ligands, the energy was minimized and converted to pdbqt format using the MGL tools of the AutoDockTools4 software [24] for further analysis. To select the preferred orientation of the ligands in the most energetically favorable pose with the complexes, we used Autodock Vina [24] at maximum exhaustiveness of 20 for blind protein–ligand docking. We identified the location of the substrate-binding site for each protein from UniProtKB [26] and catalytic site Atlas annotations [27]. The box size remained the same for all runs: 30 × 30 × 30 Å. For the analysis of the 3D visualization PyMol [28] was used.

The best interaction binding energy (kcal/mol^-1^) was selected for molecular dynamics simulations. All MD simulations were run using the AMBER20 software package [29] with the protein being assigned ff99SB [30] parameters and docked ligands being assigned GAFF [31] parameters augmented by AM1-BCC [32] partial charges. Solvation of the systems were carried out using the TIP3P [33] explicit water model and the AMBER pmemd module was used to perform energy minimizations and MD. A two-stage solvent energy minimization was performed. In the first stage, an energy minimization was performed in order to relax the solvation structure, thus restricting the protein atoms leaving it fixed in order to allow the solvent in the solvent box to move and relax around the protein, this stage consisted of 2.000 steps. In the second step, another minimization of the energy of the solvent was performed but without any restrictions to obtain the optimization of the geometry of the whole system (protein and solvent), this process consisted of 8.000 steps. Then, the thermalization of the system was performed according to the AMBER forcefield, the system was heated from 0 to 298 K gradually. A temperature ramp was generated for this purpose, which was increased until the expected temperature was achieved, using the SHAKE [34] algorithm in 10.000 steps. After heating, an equilibration for 20 ns was obtained at constant temperature and pressure (298 K and 1 Bar) to accommodate the system volume to obtain an adequate density. Finally, the well-equilibrated complexes were then subject to the production phase without any restraints for a period of 120 ns with a time step of 2 fs, and after every 2 ps the strutural coordinates were saved. The CPPTRAJ module [35] of AmberTools was used to calculate the root mean square deviation (RMSD).

#### MM-PBSA binding free energy calculation

Molecular Mechanics-Poisson Boltzmann surface area (MM-PBSA) analyses were carried out by using the MMPBSA.py [36] python script implemented in the AMBER20 package with the aim to estimate the binding free energy. The binding free energies (reported in kcal/mol) in MM-PBSA method calculate the difference between bound and unbound state of solvated conformations of a molecule, Eq. 4:

4 $$\triangle G_{\mathrm{bind},\mathrm{solv}}=\triangle G_{\mathrm{bind},\mathrm{vacuum}}+\triangle G_{\mathrm{solv},\mathrm{complex}}-\left(\triangle G_{\mathrm{solv},\mathrm{ligand}}+\triangle G_{\mathrm{solv},\mathrm{receptor}}\right)$$

The energy terms were extracted every 20 ns of each respective MD trajectory (120 ns) by selecting 1.000 uniformly spaced out snapshots.

## Results

### Chemistry

**Fig. 1:**
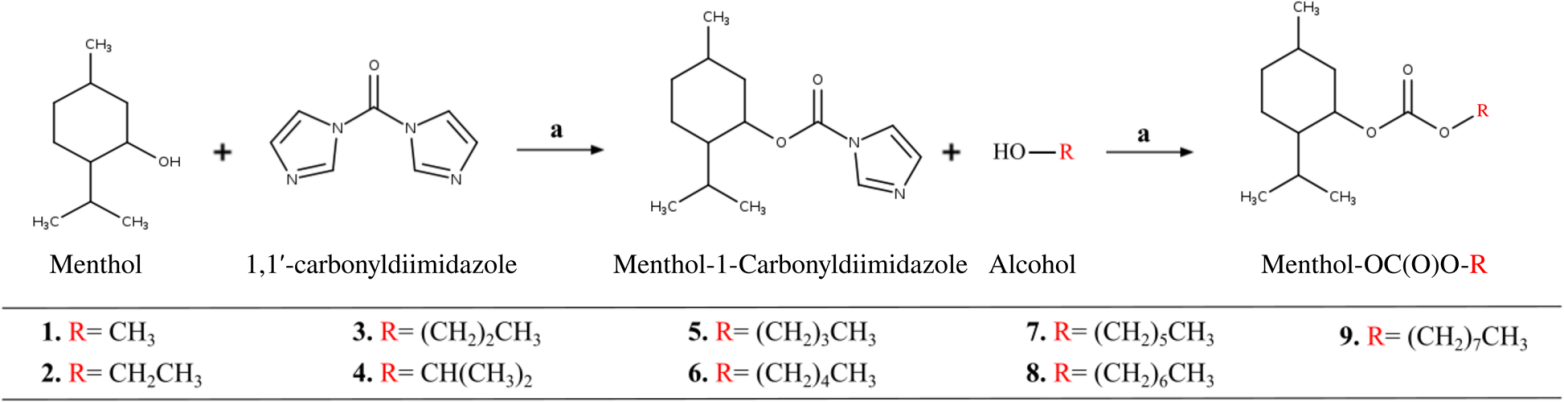
Synthesis general of menthol carbonates (**1–9**). Reagents and conditions: **a** CH_2_Cl_2_, N_2_, 25 °C, stirring, 1 h

The synthesis strategy was based on the modification of menthol hydroxyl group with different aliphatic alcohols by carbonate linkage. The new menthol derivatives obtained (**1-5,7-9**) were synthesized by a two-step method previously reported, as shown in Fig. 1 [14, 16, 17].

The first step was to obtain the carbamate intermediate (menthol-1-carbonilimidazole) by the addition of 1,1′-carbonyldiimidazole (CDI) and menthol. Stirring the carbamate intermediate with the corresponding alcohol under constant N_2_ conditions gives compound **1**–**9** with good yields (85-90%).

The IR spectra of novel derivatives obtained from menthol (**1**–**9**) showed characteristic absorption bands in the region of 1760–1778 cm^−1^ corresponding to the OC(O)O group and it is not observed the band corresponding to the hydroxyl group present in menthol (3511 cm^−1^). The ^1^H-NMR spectra of compounds (**1**–**9**) showed ring protons (H-2, H-3, H-4, H-5 and H-6) between 2.0 and 1.0 ppm. We also observed the presence of proton signals corresponding to aliphatic alcohols at 0.8–0.6. These signals correlated well with menthol and the corresponding alcohols. These results confirm the proposed structure of menthol and compounds **1**–**9**. ^13^C-NMR spectra of compounds (**1**–**9**) confirm the formation of menthol carbonates. The most significant features in ^13^C-NMR spectra of **1**–**9** were the signal at 148 ppm corresponding to the C(O) carbon. Other ^13^C-NMR signals are also in concordance with those of menthol and the corresponding aliphatic alcohol. HRMS spectra of all compounds are characterized by the presence of distinctive molecular ion peaks at the expected m/z values.

Stability studies are important requirements of prodrug screening. Therefore, regarding the chemical stability of these compounds, it is important to note that the conversion rates of compounds **1**–**9** to menthol at pHs 1.2, 5.8 and 7.4 at 37 °C were found to be independent of the length of the aliphatic chains. All of them showed a half-life of approximately 60 min at pH 1.2, while at pH 5.8 and 7.4 the half-life was higher (48 h and 24 h respectively), confirming their behavior as prodrugs (Clemente et al., unpublished).

### Biological activities

Determination of the toxicity and biological effectiveness of the prepared derivatives is one of our main objectives in this work, so we have tested all prepared derivatives against amastigotes of *T. cruzi* and *L.braziliensis*; and total forms of *P. falciparum*. Cytotoxicity activity was measured by determining the median lethal concentration (LC_50_) that corresponds to the concentration of the drug that gives the half-maximal mortality of cells. Biological effectiveness was measured by determining the median effective concentration (EC_50_) that corresponds to the concentration of the drug that gives the half-maximal reduction of the number of parasites. The results of in vitro cytotoxic and biological activity are summarized in Table 1.

**Table 1:** In vitro cytotoxicity and antiprotozoal activity of menthol and its prodrugs

Compound	Cytotoxicity	Antiplasmodial activity		Trypanocidal activity		Leishmanicidal activity	
	**LC**_**50**_** (μΜ)**^**a**^	**EC**_**50**_** (μΜ) **^**b**^	**IS**^**c**^	**EC**_**50**_** (μΜ) **^**b**^	**IS**^**c**^	**EC**_**50**_** (μΜ) **^**b**^	**IS**^**c**^
Menthol	265.7 ± 188.1	358.5 ± 51.0	0.7	173.4 ± 28.9	1.5	> 128.0	> 2.1
**1**	68.5 ± 6.7	127.4 ± 9.9	0.5	105.45 ± 34.52	0.6	22.9 ± 2.8	3.0
**2**	103.3 ± 35.8	28.4 ± 0.4	3.6	33.8 ± 4.3	3.1	28.4 ± 0.4	3.6
**3**	57.0 ± 14.4	60.7 ± 1.2	0.9	33,83 ± 2,84	1.7	31.8 ± 4.1	1.8
**4**	145.2 ± 74.5	60.7 ± 1.0	2.4	2061.7 ± 175.4	0.1	43.2 ± 0.3	3.4
**5**	207.3 ± 38.2	56.3 ± 1.0	3.7	121.7 ± 21.4	1.7	59.3 ± 1.4	3.5
**6**	221,51 ± 21,07	59.1 ± 2.6	3,7	29.6 ± 1.2	7.4	96.7 ± 0.6	2,3
**7**	73.3 ± 15.8	432.5 ± 238.7	0.2	46.3 ± 4.9	1.6	28.2 ± 1.1	2.6
**8**	48.4 ± 6.1	67.4 ± 5.8	0.7	27.6 ± 4.4	1.8	> 23.5	> 2.1
**9**	44.1 ± 3.8	62.4 ± 6.5	0.7	26.7 ± 4.6	1.7	463.3 ± 205.7	0.1
DOXO^d^	1.7 ± 0.2	NA^e^	NA	NA	NA	NA	NA
CQ^f^	485.3 ± 16.3	10.5 ± 1.3	46.2	NA	NA	NA	NA
BNZ^g^	> 768.5	NA	NA	60.1 ± 12.2	12.8	NA	NA
AMB^h^	53.9 ± 7.4	NA	NA	NA	NA	0.4 ± 0.1	134.8

Data represent the lethal and effective concentration for each compound

^a^Median lethal concentration

^b^Median effective concentration

^c^*IS* Index of Selectivity = LC_50_/EC_50_

^d^*DOXO* Doxorubicin

^e^*NA* No apply, *CQ* Cloroquine, *BNZ* Benznidazole, *AMB* Amphotericin B

### Computational chemistry

#### Drug-likeness

The aim of calculating drug-likeness profiles is to provide, with reasonable accuracy, a preliminary prediction of the in vivo behavior of a compound to assess its potential to become a drug. The prodrugs used in this study were subjected to the calculation of their absorption, distribution, metabolism, and excretion (ADME) properties. In addition, physicochemical properties, such as molecular hydrogen bond acceptor (HBA), hydrogen bond donor (HBD), molecular weight (MW), topological polar surface area (TPSA), number of rotational bonds (RB), octanol/water partition coefficient (LogP), and molar refractivity (MR), using the SwissADME web server [22]. The toxicological properties of the compounds were analyzed taking into account the empirical toxicity rules of Lipinski [37], Ghose [38] and Veber [39].

To evaluate if the designed molecules can be selected as potential drugs we predicted some pharmacokinetic properties (Table 2 and Figure S1). From this, it was corroborated if the compounds comply with the rules of Lipinski, Ghose and Veber. If any of the compounds only satisfied two of the three rules, we took it as a precaution; if it satisfied only one rule, that molecule was not a good candidate.

**Table 2:** ADME molecular descriptors of compounds designed

Compound	MW (g/mol)	LogP	HBA	HBD	TPSA(Å^2^)	RB	MR	RL	GR	VR	Synth. Acce
Menthol	156.27	2.58	1	1	20.23	1	49.23	Yes	Yes	Yes	2.63
1	214.31	3.04	3	0	35.54	4	60.44	Yes	Yes	Yes	3.49
2	228.33	3.33	3	0	35.54	5	65.25	Yes	Yes	Yes	3.64
3	242.36	3.74	3	0	35.54	6	70.06	Yes	Yes	Yes	3.75
4	242.36	3.64	3	0	35.54	5	70.06	Yes	Yes	Yes	3.75
5	256.39	3.98	3	0	35.54	7	74.86	Yes	Yes	Yes	3.86
6	270.41	4.42	3	0	35.54	8	79.67	Yes	Yes	Yes	3.97
7	284.44	4.68	3	0	35.54	9	84.48	Yes	Yes	Yes	4.08
8	298.47	5.15	3	0	35.54	10	89.28	Yes	Yes	Yes	4.20
9	312.49	5.39	3	0	35.54	11	94.09	Yes	Yes	No	4.31

*MW* Molecular weight, *LogP* octanol/water partition coefficient, *HBA* Hydrogen Bond Acceptor, *HBD* Hydrogen Bond Donor, *TPSA* Topological Polar Surface Area, *RB*: Rotatable Bond, and *MR* Molar Refractivity. *LR* Lipinski Rules, *GR* Ghose Rules, *VR* Veber Rules, and Synth. *Acce*. Synthetic accessibility

According to the results shown in Table 2, it is observed that all the prodrugs met the expected values for the Lipinski parameters. Therefore, their bioavailability and absorption are not deficient, and they are expected to be orally active. Similarly, all compounds complied with Ghose’s rule. With respect to Veber’s rule, all of them are fulfilled except compound **9** because it has 11 rotatable bonds and the rule allows up to 10 rotatable bonds.

#### Molecular docking and dynamics studies

Pyrimidines are essential for cell survival and proliferation in parasitic organisms such as *T. cruzi*, *L. braziliensis* and *P. falciparum*. In human cells, pyrimidines are synthesized through de novo or salvage biosynthesis pathways, which is an efficient way to recycle pre-existing nucleotides. Due to the fact that a large number of parasitic organisms lack pyrimidine rescue pathways for pyrimidine nucleotides, blocking de novo biosynthesis is considered an effective therapeutic strategy to selectively target the parasite without affecting the human host [40]. Dihydroorotate dehydrogenase (DHODH) is emerging as a new molecular target of interest for antiparasitic drugs against neglected infectious diseases [40–42]. This protein is the only redox enzyme of the six involved in the de novo biosynthetic pathway of pyrimidines; this protein catalyzes the oxidation of (S)-dihydroorotate to orotate.

Due to the mentioned and several reports indicating that natural compounds with structures similar to enzyme substrates of parasite biosynthetic pathways could exhibit inhibitory properties [42–46]; we report the results of molecular docking and molecular dynamics simulations to analyze the binding energies and intermolecular interactions between menthol and TcDHODH, LbDHODH and PfDHODH. The compounds designed, synthesized, characterized and evaluated (**1**–**9**) are defined as prodrugs because the carbonate linkers are susceptible to be hydrolyzed chemically and by enzymes present in the organism [47–50], for this reason the in silico studies were carried out with the parent compound.

**Fig. 2:**
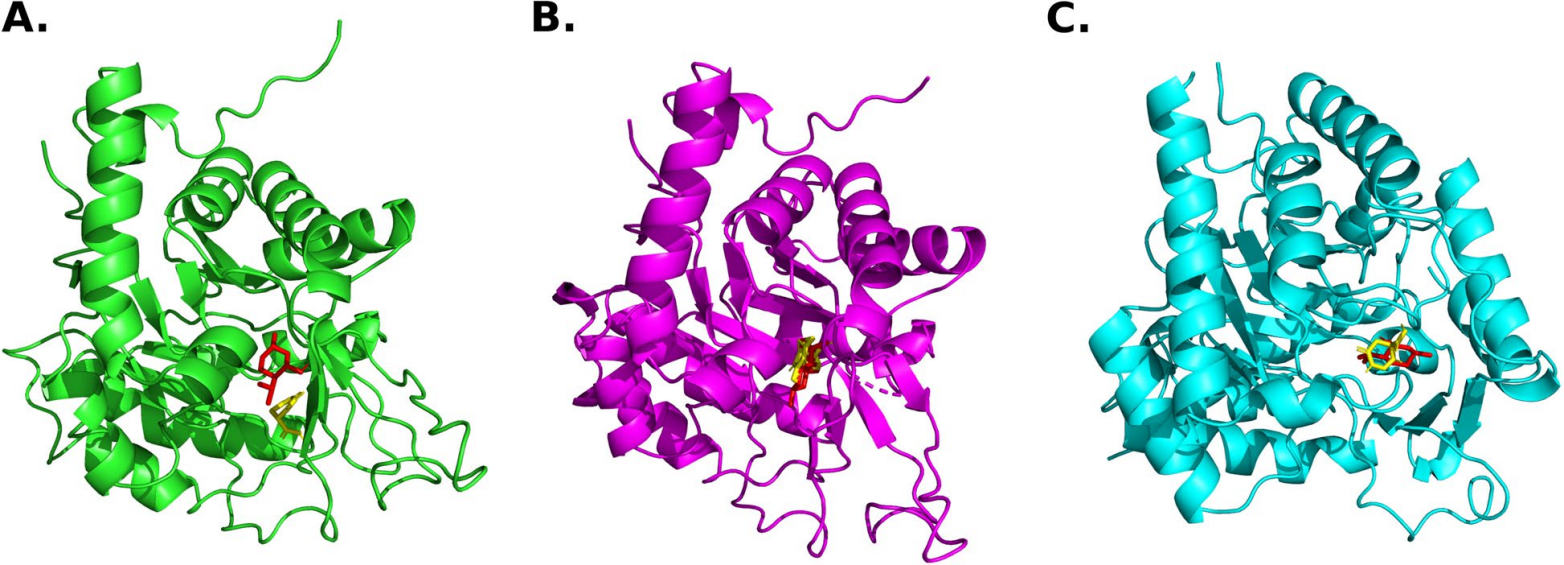
Predicted binding modes of menthol and natural substrate in complex with (**A)** TcDHODH, (**B)** LbDHODH and (**C)** PfDHODH

Following validation of AutodockVina (see Material and Methods), menthol and natural substrate were docked to the substrate-binding site of TcDHODH, LbDHODH and PfDHODH. The most energetically favorable conformation of each ligand was selected to form the protein–ligand complex that will subsequently be submitted to molecular dynamics simulations (Fig. 2).

Molecular dynamics (MD) simulations will allow us to observe the dynamic behavior of the complexes, analyzing the stability of the complex and its interactions. MD simulation studies of 120 ns for each complex were carried out. The trajectories obtained for simulations were analyzed using RMSD, and ligand–protein interactions in terms of binding free energy calculations (MM-PBSA).

**Fig. 3:**
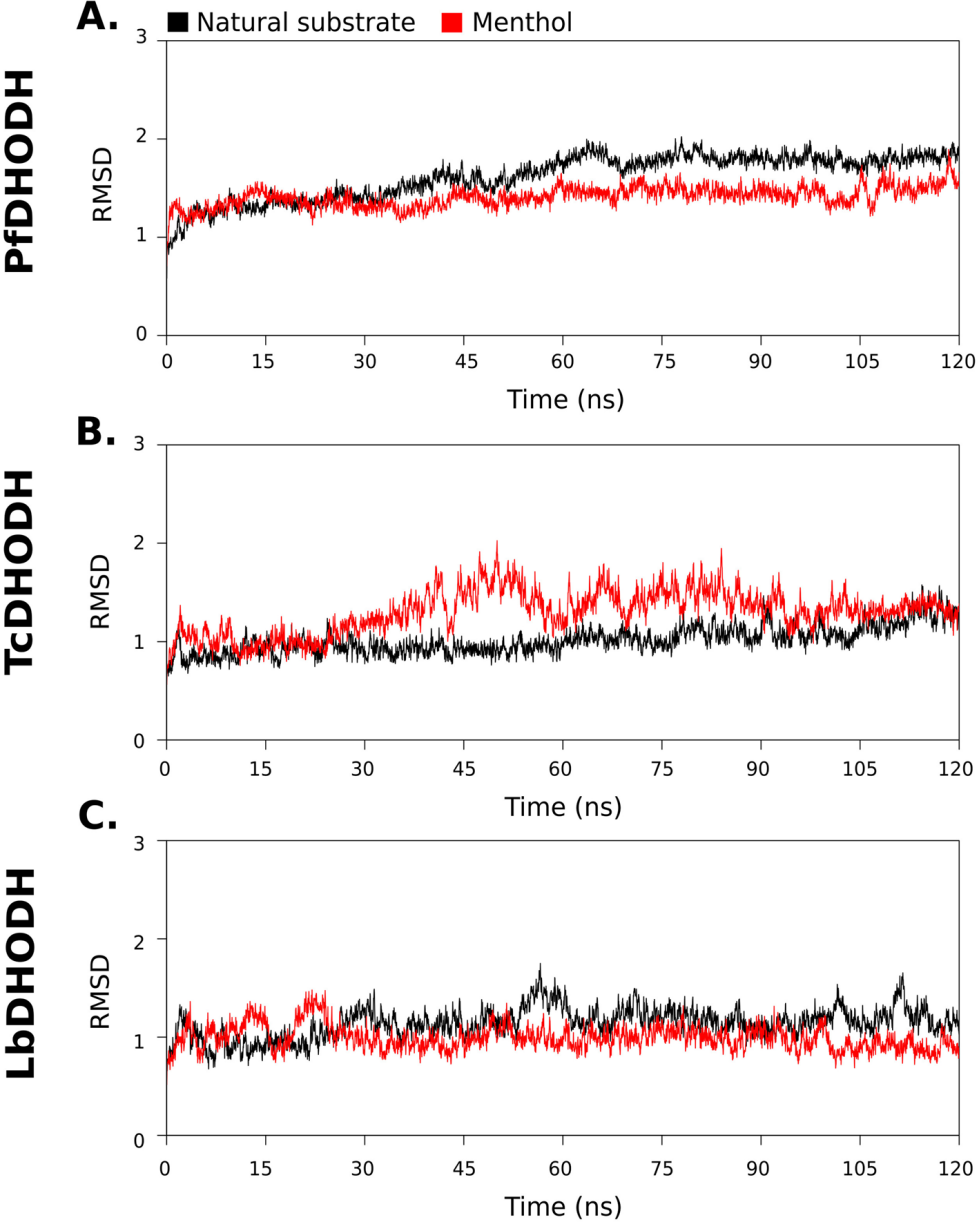
RMSD of the backbone atoms of the docked complexes. In red menthol complexes and in black natural substrate complexes

Root mean square deviation (RMSD), is a useful parameter to quantify the overall structural stability of a ligand–protein complex after binding of the ligand within substrate-binding site, in the function of a period of time. It can be seen in the RMSD protein graphs (Fig. 3 A-C) that the three complexes analyzed show deviations within the RMSD range of 0.5 Å to 2.0 Å during the whole simulations. Values up 3–4 Å are completely satisfactory for globular proteins, indicating that their bindings are significantly stable in all the analyzed systems.

The average RMSD values of the protein backbone in the simulated complexes for natural substrate and menthol in PfDHODH were 1.20 and 1.27, in TcDHODH 0.85 and 1.01, in LbDHODH were 0.95 and 0.99, respectively. Based on these average RMSD values, we can clearly infer those complexes with menthol are conformational stable since they have close values to the complexes simulated with the natural substrate. It was noticed that in the TcDHODH-Menthol complex there is a small difference with respect to the behavior with the natural substrate, however the RMSD values are still significantly stable.

#### MM-PBSA binding free energy calculation

As the docking revealed some clear trends in the type of interaction, the MD simulations provided a more detailed understanding of the nature, strength and persistence of interactions with key residues. The binding free energy (MM-PBSA) was carried out on all complexes to predict their binding affinities, the results are given in Table 3.

**Table 3:** Predicted binding free energies (kcal/mol^−1^) and individual energy terms, calculated from molecular dynamics simulation through the MM/PBSA protocol for TcDHODH, LbDHODH and PfDHODH complexes

Ligand	Energy Component (kcal/mol^−1^)				
	*ΔEvdw*	*ΔEle*	*ΔGgas*	*ΔGsol*	*ΔGtotal*
**TcDHODH**					
(S)-dihydroorotate	-17.41 ± 2.00	-17.18 ± 3.94	-34.60 ± 4.46	25.42 ± 4.22	-9.17 ± 3.44
Menthol	-22.33 ± 1.44	-4.65 ± 3.45	-26.99 ± 2.54	16.37 ± 3.42	-10.62 ± 3.63
**LbDHODH**					
(S)-dihydroorotate	-20.44 ± 1.98	-27.20 ± 3.18	-47.65 ± 3.05	33.76 ± 2.21	-13.88 ± 0.34
Menthol	-26.22 ± 1.23	-1.45 ± 0.73	-27.68 ± 1.49	19.19 ± 276	-8.48 ± 2.62
**PfDHODH**					
(S)-dihydroorotate	-18.63 ± 2.98	-60.50 ± 5.21	-79.14 ± 4.00	50.44 ± 2.69	-28.69 ± 3.65
Menthol	-26.58 ± 1.83	-13.65 ± 1.54	-40.23 ± 1.85	19.13 ± 1.74	-21.10 ± 2.06

*ΔEvdw* Van der Waals energy, *Δele* Electrostatic energy, *ΔGgas* Gas-phase free energy, *Δgsol* Solvation free energy., *Δgtotal* Total binding free energy

All simulated complexes obtained favorable binding energy, this suggests that menthol would be able to bind favorably to TcDHODH, LbDHODH and PfDHODH. TcDHODH-Menthol complex obtained a higher binding energy than the TcDHODH-(S)-dihydroorotate complex, -10.62 kcal/mol^-1^ and -9.17 kcal/mol^-1^ respectively. Furthermore, this could indicate that in the presence of both (S)-dihydroorotate and menthol, the latter may have a higher binding affinity for the substrate binding site.

Several components of the binding free energy were analyzed to identify the mechanisms of interaction of these molecules with the substrate binding site, as well as the relevant interactions that explain in particular their binding specificity. In all complexes the energy of intermolecular interactions of the gas phase (nonpolar solvation) was the most important contribution to binding. Favorable interactions with respect to van der Waals energy and electrostatic energy were also recorded in all cases. On the other hand, these favorable contributions were counteracted by the polar solvation energy.

The energetic decay of the *Δ*G was calculated in terms of the contribution of each substrate binding site residue in all complexes over the 120 ns of the molecular dynamics simulations (Figure S2). The main favorable interactions originated predominantly at residues contributing binding energies greater than -0.5 kcal/mol. From the complex with TcDHODH-Menthol it was observed that the predominant energetic contributions of Menthol were Ala19 (-0.72 kcal/mol), Leu22 (-0.65 kcal/mol), Ser195 (-0.80 kcal/mol) and Ala273 (-0.73 kcal/mol). We highlight the contribution of the Ser195 residue as it was reported as a residue that could inhibit the protein through its interaction [51]. With respect to the LbDHODH-menthol complex, the following favorable energetic contributions were observed: Ala19 (-0.82 kcal/mol), Val22 (-1.14 kcal/mol) and Thr273 (-0.80 kcal/mol). In terms of the PfDHODH-Menthol complex, the following favorable energetic contributions were identified Leu172 (-1.03 kcal/mol), Phe188 (-0.91 kcal/mol), Ile272 (-0.93 kcal/mol), Ser529 (-1.32 kcal/mol) and Val532 (-1.29 kcal/mol). The interaction found with Leu172 and Phe188 is significant because Booker et al. reported that different PfDHODH inhibitors generated interaction with these residues [52].

## Discussion

Despite the high impact of Chagas, Leishmaniasis and Malaria diseases on morbidity and mortality, there are still no treatments with high efficacy and low toxicity for the patient. In this study, we evaluated the in vitro and in silico antiparasitic activity of new menthol prodrugs against *T. cruzi*, *L. braziliensis* and *P. falciparum*.

Only compounds **8** and **9** were shown to be cytotoxic to U-937 cells, with LC_50_ values of 48.4 µM and 44.1 µM, respectively; this observed cytotoxicity is caused by compounds with more lipophilic carbon chains. Also as expected, DOXO showed high cytotoxicity (1.7 µM). The remaining compounds showed moderate to low cytotoxicity.

The dose–response relationship significantly revealed that the majority of the compounds were highly active against intracellular amastigotes of *T. cruzi* and *L. braziliensis*. Compound **2** was one of the drugs that showed a high SI for the three organisms evaluated.

For *T. cruzi*, we observed that compounds **2**, **6–9** showed high trypanocidal activity from 26.7 to 46.3 µM, while menthol obtained a low activity with an EC_50_ of 173.4 µM. BNZ, used as a control compound for antitrypanocidal activity, showed a EC_50_ of 60.1 µM. Therefore, the aforementioned compounds with high activity were not only more active than the parent compound but also more active than the reference drug used for the treatment of *T. cruzi*.

In addition, compounds **1**–**4,** and **7** demonstrated promising high activity against intracellular amastigotes of *L. braziliensis*, with EC_50_ values of 22.9 µM and 43.2 µM. Compounds **5** and **6** showed moderate activity with EC_50_ values of 53.3 µM and 96.7 µM, respectively. Although several menthol prodrugs are reported to exhibit high antileishmanial activity, none was more active than AMB with an EC_50_ 0.4 µM. Silva et al. (2017) reported that menthol had a weaker antiparasitic action against *Leishmania amazonensis* promastigotes compared to the phenolic compound eugenol (EC_50_ 1272.87 uM and 504.87 respectively) [53]. Analyzing previous studies, we found a similar trend to that reported by Silva, as the EC_50_ reported for *L. branziliensi*s of menthol was higher than 128 and that of eugenol was 60.4 [53].

In terms of antiplasmodial activity, it was found that only compound **2** and CQ were shown to have high activity, with EC_50_ values of 28.4 µM and 10.2 µM, respectively. The rest of the compounds (**3**, **4**–**6**, **8,** and **9**) showed moderate activity from 56.3 to 67.4 µM. It is highlighted that these compounds with high and moderate activity resulted in better antiplasmodial activity than the starting compound menthol with an EC_50_ 358.5 µM. In contrast, compounds **1** and **7** showed low antiplasmodial activity.

To observe the relationship between cytotoxicity and the biological activity reported, the SI was used as an indicator, which corresponds to the coefficient between the EC_50_ in the different stages of the parasite and the LC_50_ in U-937 cells. Compounds with a selectivity index of 3 or more were considered selective. It was observed that the compounds **2**, **5** and **6** were selective for *P. falciparum*. For *T. cruzi*, compounds **2** y **6** were selective, compound **6** was the most selective with an IS of 7.4. In the case of *L. braziliensis*, compounds **1**, **2**, **4** and **5** were selective.

In other studies, Fabbri et al. evaluated the in vitro efficacy against *E. multilocularis* protoscolices and the in vivo efficacy against the murine model of alveolar echinococcosis. Compound **6** was found to have a greater protoscolicidal effect than menthol. In addition, the novel compound demonstrated similar clinical efficacy to albendazole. Thus, compound **6** was defined as a promising candidate as a potential alternative for the treatment of alveolar echinococcosis [14].

Based on the results obtained, the use of a carbonate bond at the -OH position of menthol combined with the use of *n*-alcohols appears to be a promising strategy to improve the potency and safety of this monoterpene.

In terms of the ADME property predictions performed, all the new compounds are found to have drug-like molecular properties and the probability to be lead candidates. Furthermore, the prediction of drug-likeness properties showed the potential of all the synthesized compounds to be orally active candidates. The above results suggest that the compounds synthesized in this work are promising and should be further evaluated in in vivo studies.

Molecular dynamics simulations and MM-PBSA binding free energy calculation performed with the TcDHODH, LbDHODH and PfDHODH proteins revealed that menthol is stable in the structural conformation of the proteins in the same order as the natural substrate. It was also predicted that the parent compound has a higher binding energy than the natural substrate in binding to the substrate binding site of TcDHODH. Finally, interactions of menthol with residues involved in the inhibition of TcDHODH and PfDHODH proteins were predicted; furthermore, these results should be experimentally verified through enzymatic assays.

## Conclusion

In the present study, the synthesis, characterization, evaluation of antiparasitic activity and prediction of the possible mechanism of action of menthol prodrugs were performed. Compounds **1**–**9** showed promising in vitro activity against intracellular amastigotes of *L. braziliensis* and *T. cruzi*; regarding the activity against *P. falciparum* the compounds presented moderate antiplasmodial activity. Furthermore, the prediction of drug-likeness properties showed the potential of all the synthesized compounds to be orally active candidates. The above results suggest that the compounds synthesized in this work are promising and should be further evaluated in in vivo studies.

## Supplementary information

**Additional file 1:** **Fig. S1.** Analysis of six physicochemical properties (lipophilicity, size, polarity, solubility, flexibility, and saturation) using bioavailability radar plot representations. The shaded area represents the range of properties to be considered drug-like. Thered line represents the properties of the test molecules.

**Additional file 2:** **Fig. S2.** Decomposition of the MD free energy of binding in terms of per residue contribution. Residues showing the most negative peaks correspond to stronger stabilizations.

A**dditional file 3.** Menthol carbonates as potent antiparasitic agents: synthesis and *in vitro *studies along with computer-aided approaches.

## Data Availability

All analyzed data during this research are included in the published manuscript. The generated datasets during this research is not publicly available though it can be providable from the corresponding author upon reasonable request.

## Abbreviations

SI: Selectivity Index
TcDHODH: *Trypanosoma cruzi* Dihydroorotate Dehydrogenase
LbDHODH: *Leishmania braziliensis* Dihydroorotate Dehydrogenase
PfDHODH: *Plasmodium falciparum* Dihydroorotate Dehydrogenase
TLC: Thin-layer Chromatography
CDI: 1,1-Carbonyldiimidazole
NMR: Nuclear Magnetic Resonance
J: Coupling Constants
Hz: Hertz
HRMS: High-Resolution Accurate Mass
FTIR: Fourier Transform Infrared Spectroscopy
FBC: Fetal Bovine Serum
DOXO: Doxorubicin
CQ: Chloroquine
AMB: Amphotericin B
BZN: Benznidazole
PMA: Phorbol Myristate Acetate
CPRG: Chlorophenol red-β-D-galactopyranoside
PBS: Phosphate Buffer
MFI: Fluorescence intensity
PDB: Protein Data Bank
RMSD: Root Mean Square Deviation
MM-PBSA: Molecular Mechanics-Poisson Boltzmann Surface Area
ADMET: Absorption, Distribution, Metabolis and Excretion (ADME)
HBA: Molecular Hydrogen Bond Acceptor
HBD: Hydrogen Bond Donor
MW: Molecular Weight
TPSA: Topological Polar Surface Area
LogP: Octanol/water partition coefficient
RB: Number of rotational bonds
MR: Refractivity
LR: Lipinski Rules
GR: Ghose Rules
VR: Veber Rules
Synth. Acce: Synthetic accessibility
MD: Molecular Dynamics
