# Supplementary figures and images for: Menthol carbonates as potent antiparasitic agents: synthesis and in vitro studies along with computer-aided approaches
Source: BMC Complement Med Ther. 2022 Jun 13;22:156. doi: 10.1186/s12906-022-03636-8 (PMC9190099; doi:10.1186/s12906-022-03636-8)

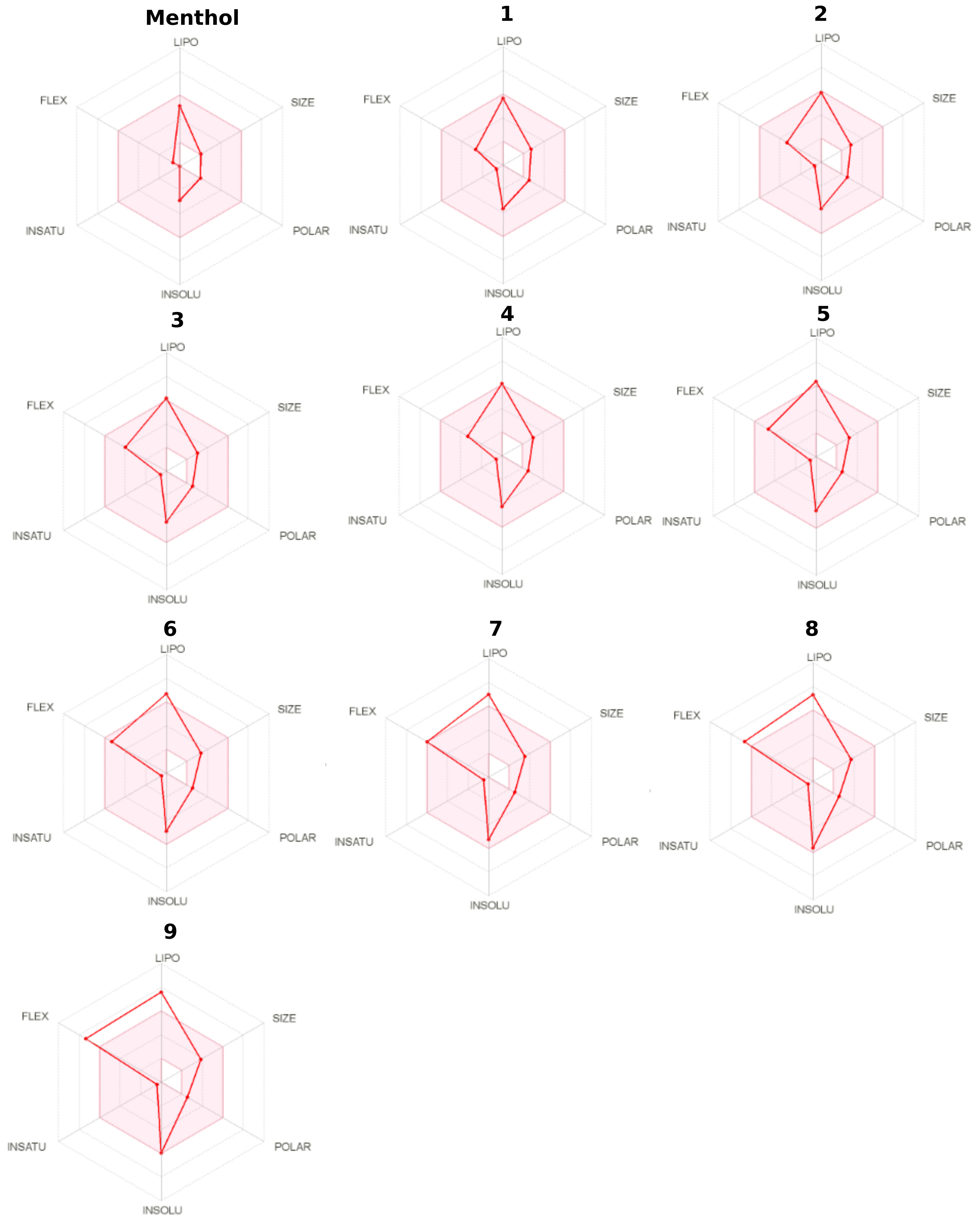

Supplement: Supplementary file 1 — Additional file 1: Fig. S1. Analysis of six physicochemical properties (lipophilicity, size, polarity, solubility, flexibility, and saturation) using bioavailability radar plot representations. The shaded area represents the range of properties to be considered drug-like. Thered line represents the properties of the test molecules. [file 12906_2022_3636_MOESM1_ESM.jpg]

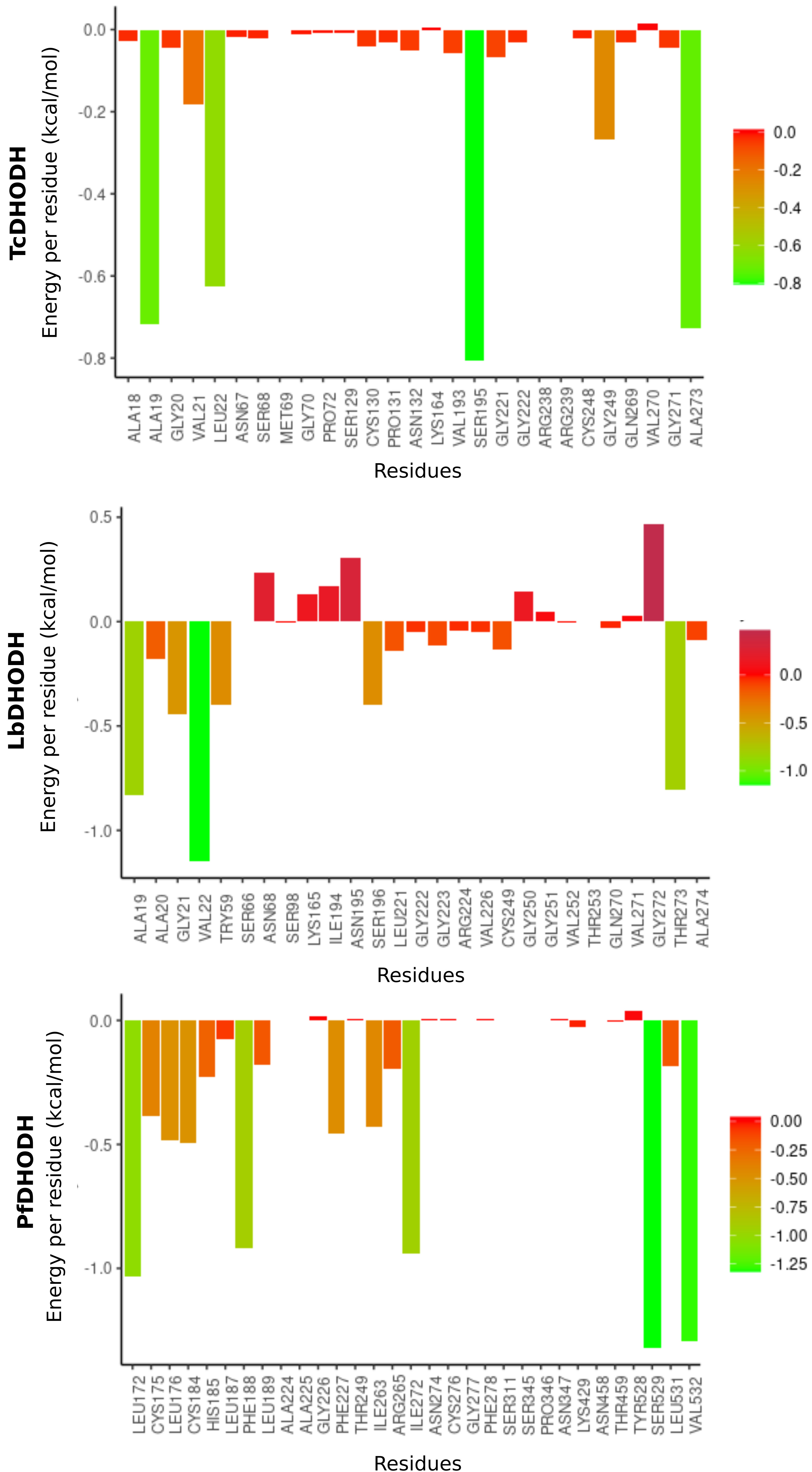

Supplement: Supplementary file 2 — Additional file 2: Fig. S2. Decomposition of the MD free energy of binding in terms of per residue contribution. Residues showing the most negative peaks correspond to stronger stabilizations. [file 12906_2022_3636_MOESM2_ESM.jpg]
