# Supplementary material for: Menthol carbonates as potent antiparasitic agents: synthesis and in vitro studies along with computer-aided approaches
Source: BMC Complement Med Ther. 2022 Jun 13;22:156. doi: 10.1186/s12906-022-03636-8 (PMC9190099; doi:10.1186/s12906-022-03636-8)
Supplement: Supplementary file 3 — Additional file 3. Menthol carbonates as potent antiparasitic agents: synthesis and in vitro studies along with computer-aided approaches. [file 12906_2022_3636_MOESM3_ESM.doc]

**Menthol carbonates as potent antiparasitic agents: synthesis and *in vitro* studies along with computer-aided approaches.**

Camila M. Clemente^1^, Sara M. Robledo^2^ and Soledad Ravetti^3*^.

^1^Instituto Multidisciplinario de Investigación y Transferencia Agroalimentaria y Biotecnológica (IMITAB). Instituto Académico Pedagógico de Ciencias Básicas y Aplicadas, Universidad Nacional de Villa María, Córdoba, Argentina.

^2^ PECET-Facultad de Medicina, Universidad de Antioquia, Medellín, Colombia

^3^Centro de Investigaciones y Transferencia de Villa María (CIT VM). Instituto Académico Pedagógico de Ciencias Humanas. Universidad Nacional de Villa María, Córdoba, Argentina.

*** Correspondence:**

Soledad Ravetti, CIT VM, Universidad Nacional de Villa María, Arturo Jauretche 1555, X5900 Villa María, Córdoba, Argentina.

Email: [sravetti@unvm.edu.ar](mailto:sravetti@unvm.edu.ar)

**
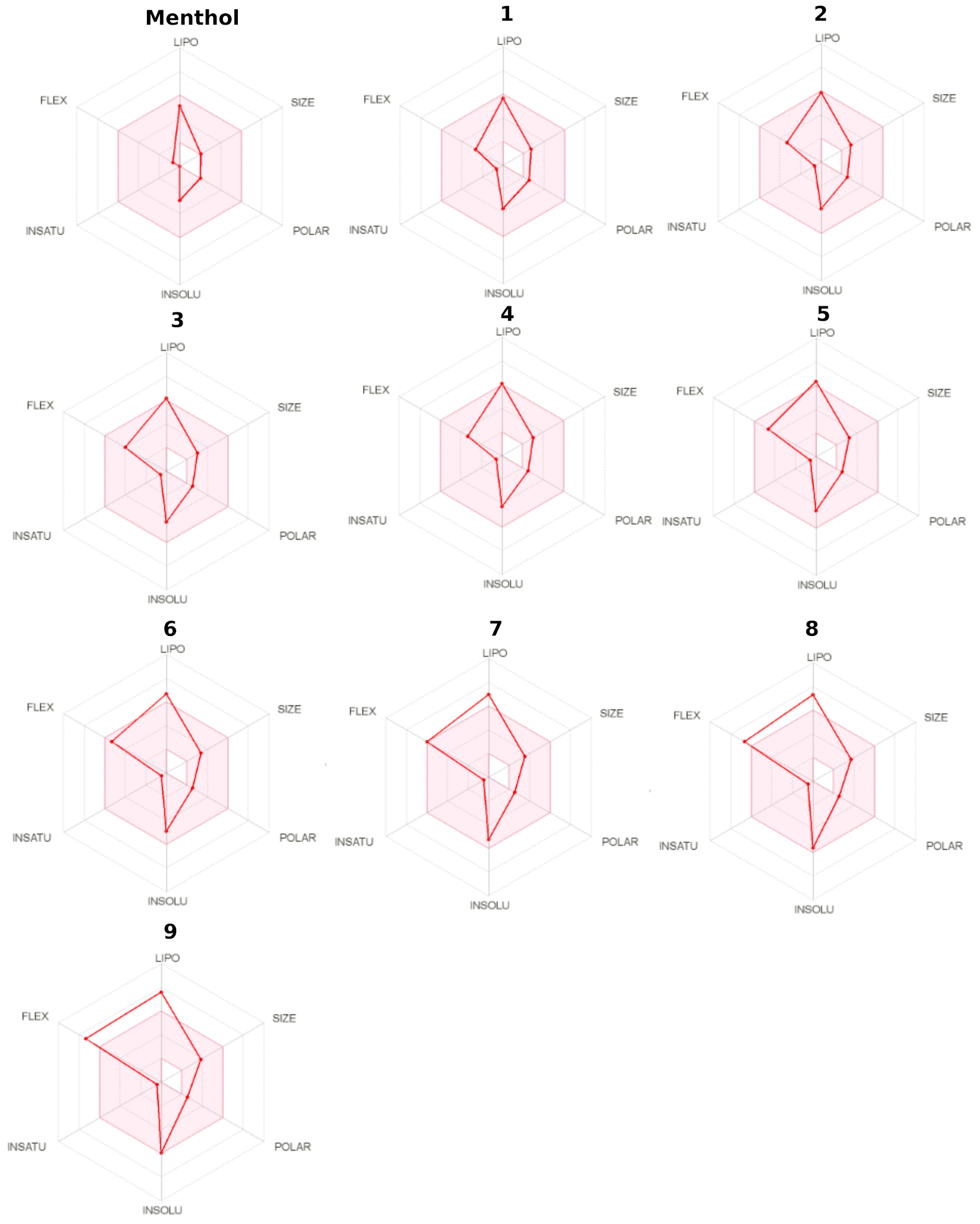
**

**Figure S1.**  Analysis of six physicochemical properties (lipophilicity, size, polarity, solubility, flexibility, and saturation) using bioavailability radar plot representations. The shaded area represents the range of properties to be considered drug-like. Thered line represents the properties of the test molecules.

Figure S2.
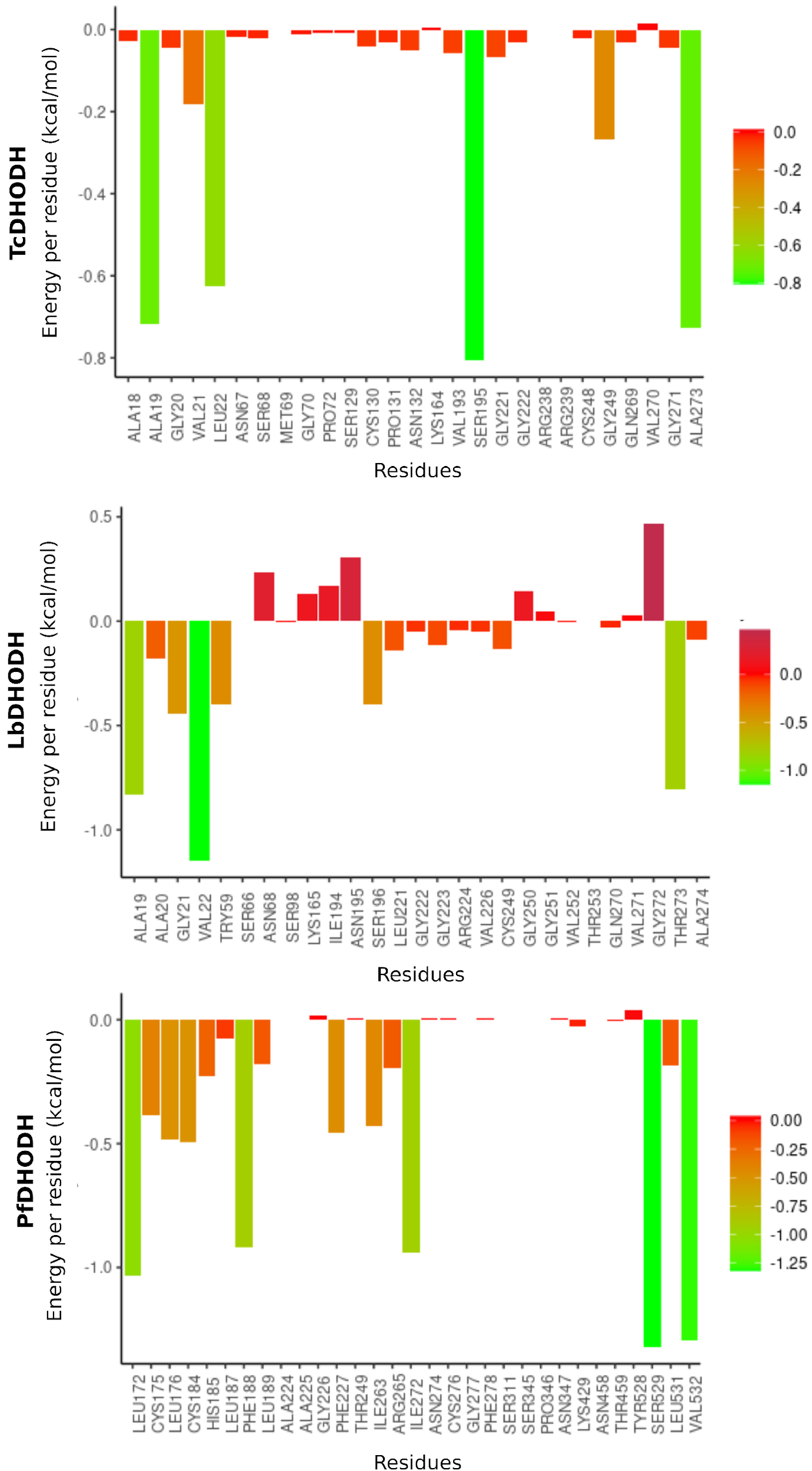
Decomposition of the MD free energy of binding in terms of per residue contribution. Residues showing the most negative peaks correspond to stronger stabilizations.


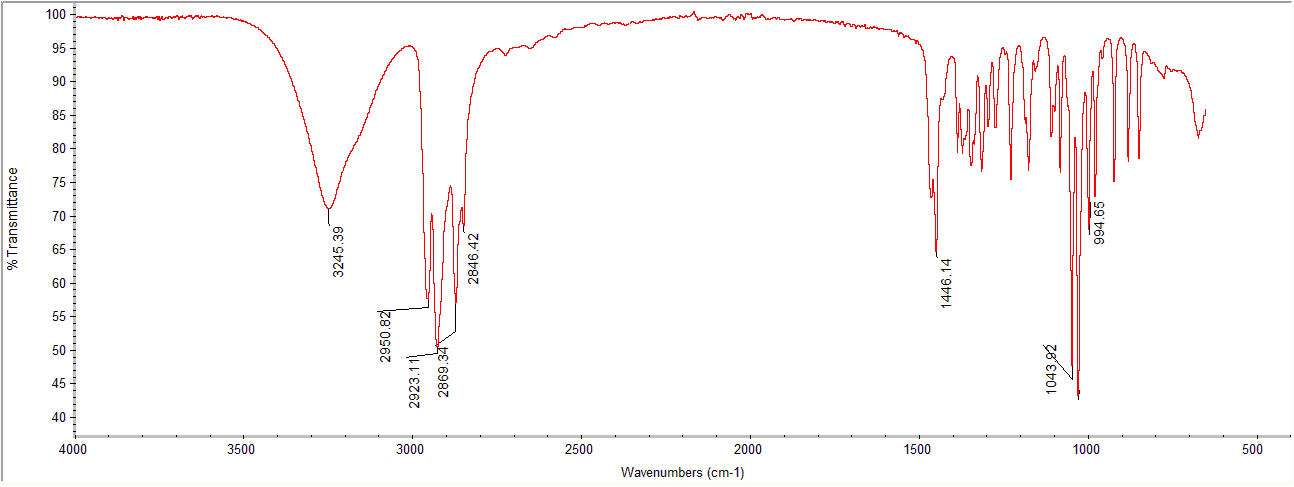


**Figure S3**. IR-FTIR spectra of Menthol

Figure S
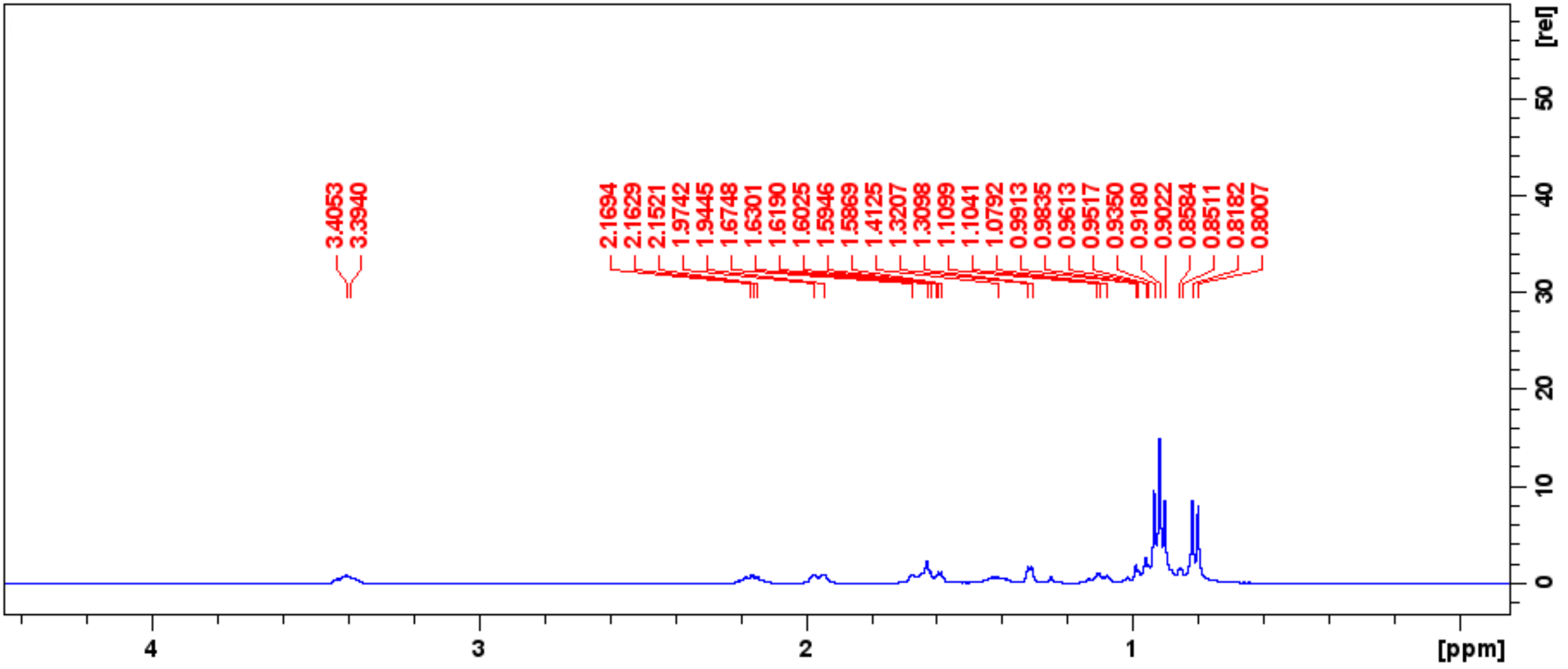
**4.** ^1^H-RMN spectra of Menthol

**
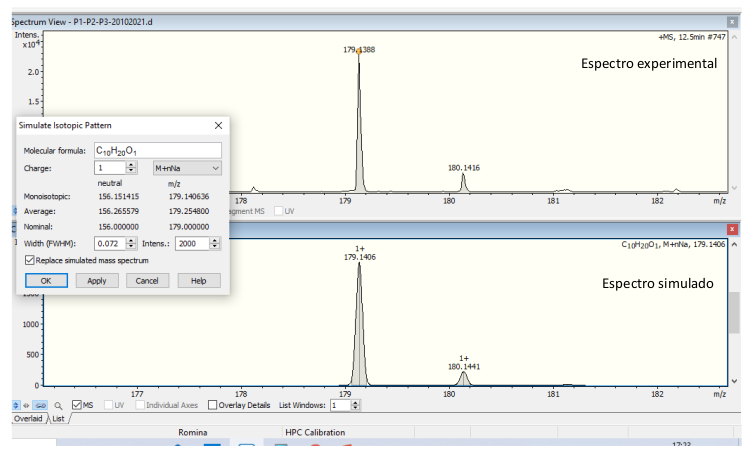
**

**Figure S5**. HRMS spectra of Menthol


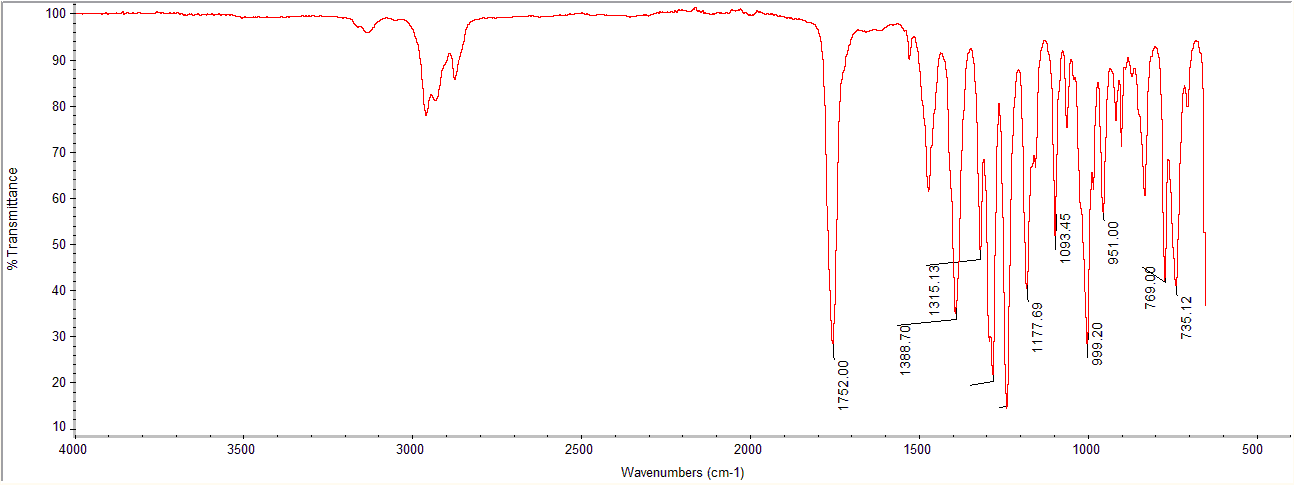


**Figure S6.** IR-FTIR spectra of compound **1**

**Figure S**^7^. 1H-RMN Spectra of compound
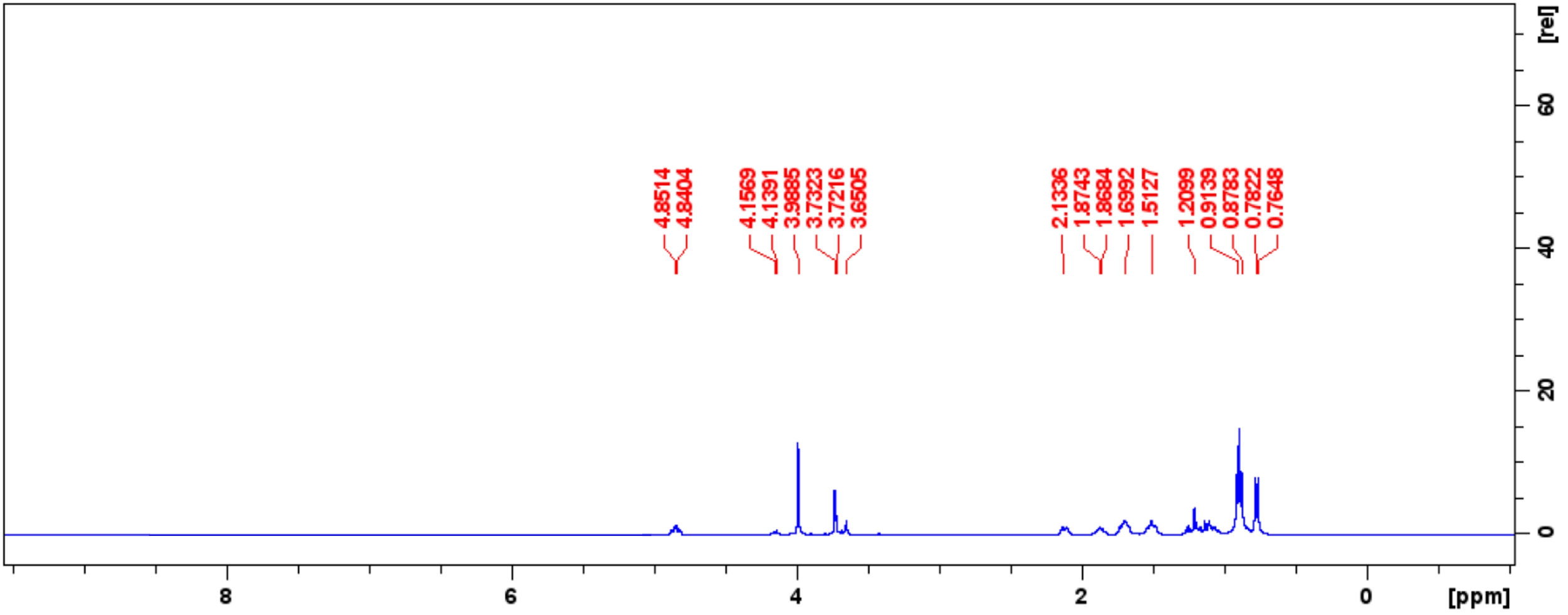

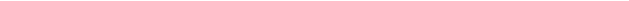
 **1**


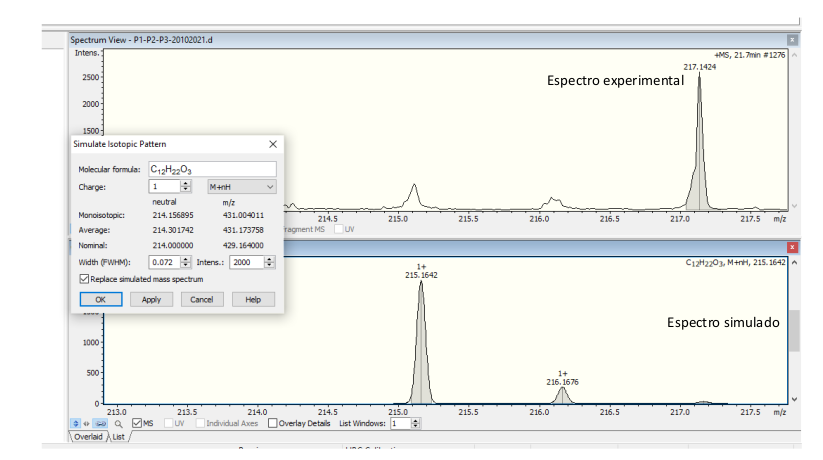


**Figure S8**. HRMS spectra of compound **1**


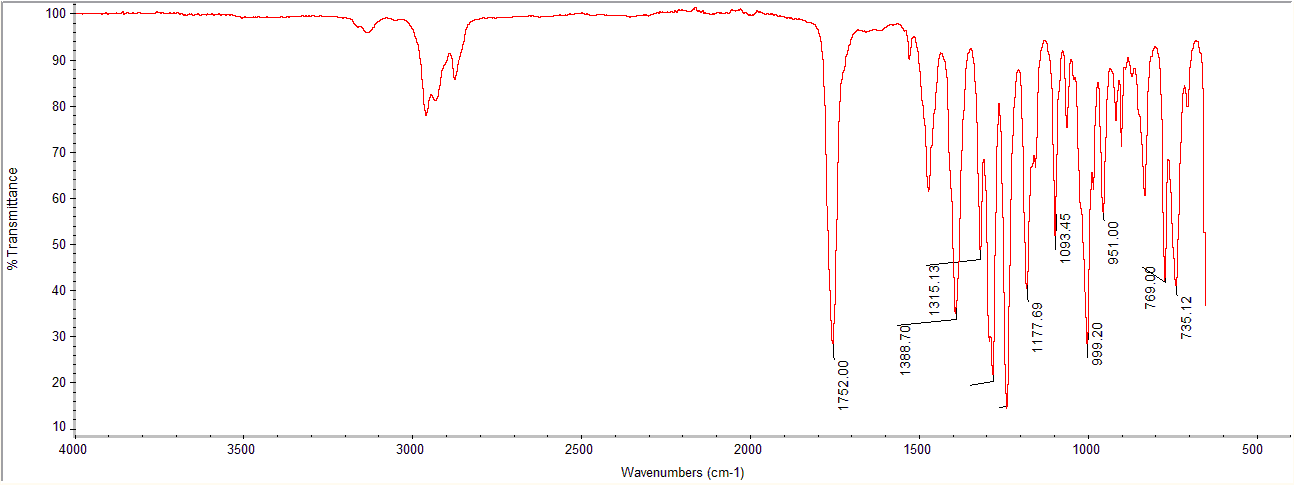


**Figure S9**. IR-FTIR spectra of compound **2**

**Figure S1**^0^. 1H-RMN spectra of compound
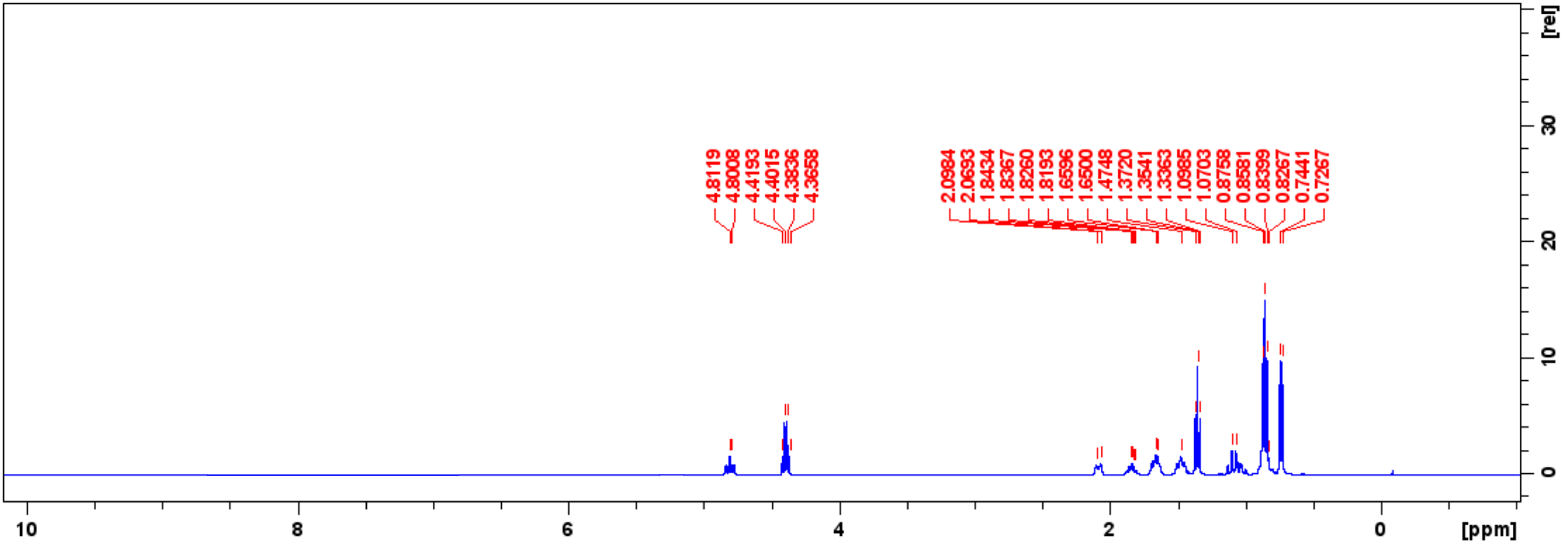
 **2**

Figure S11.
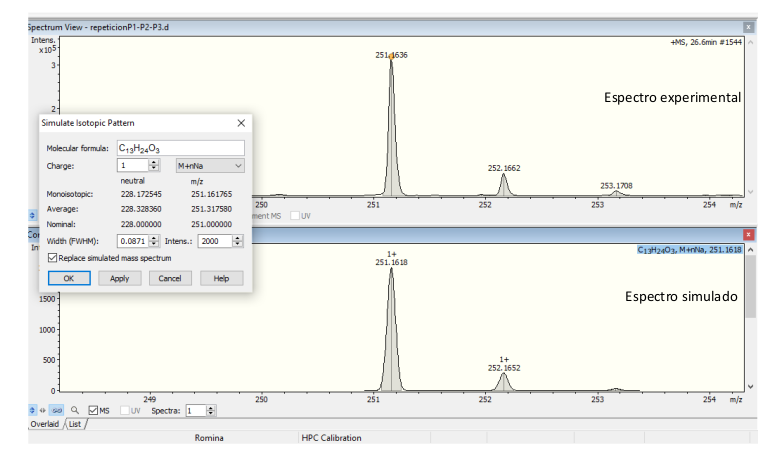
HRMS spectra of compound


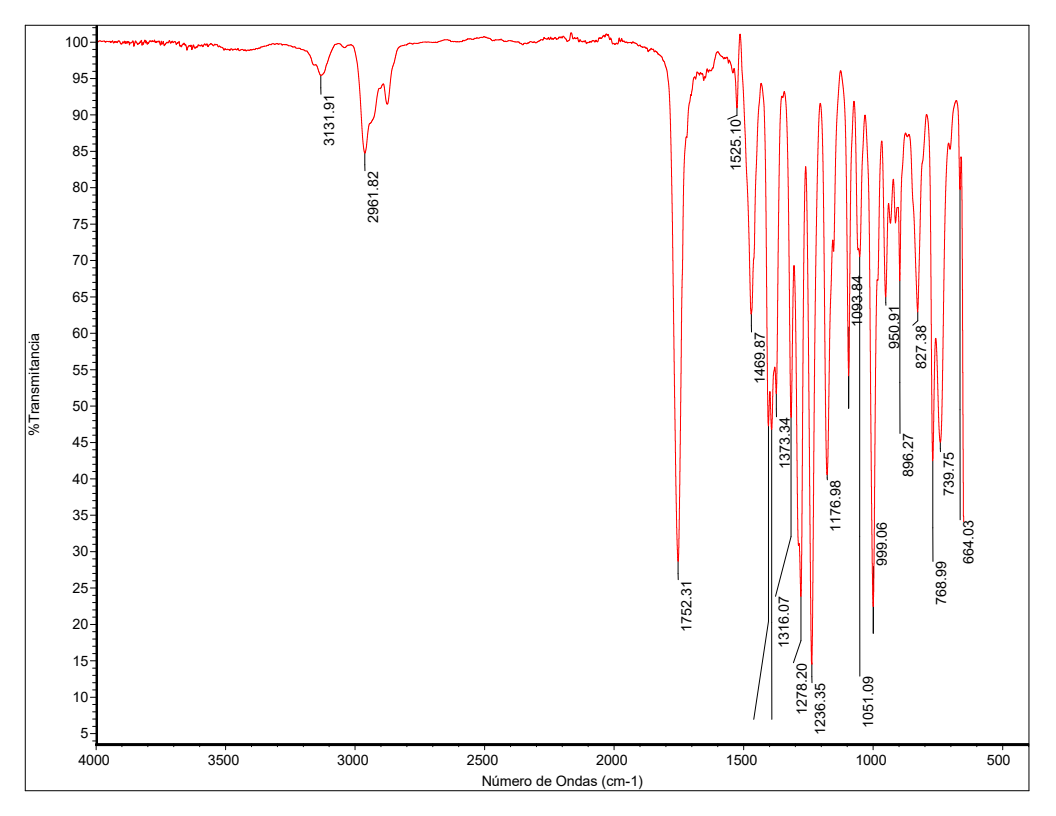
2

**Figure S12**. IR-FTIR spectra of compound **3**

**Figure S13.**. 1H-RMN spectra of
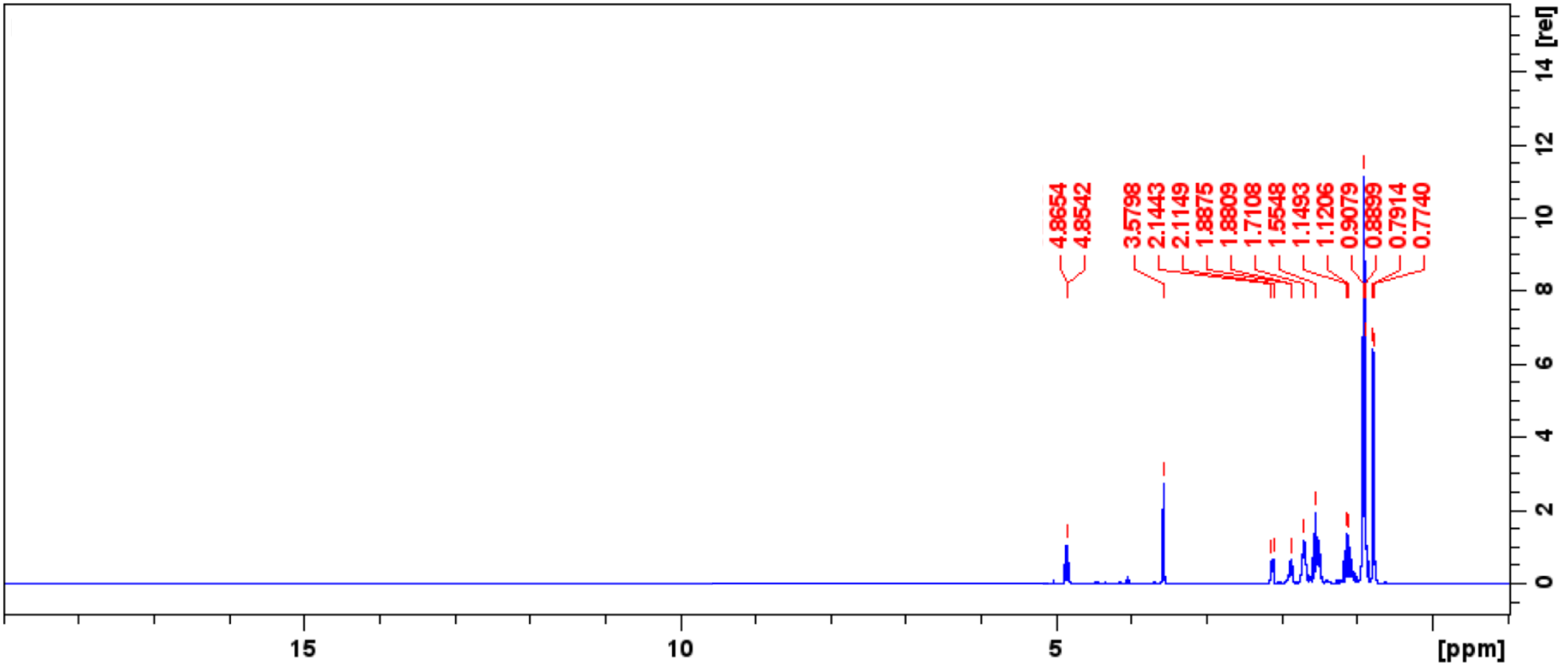
compound

**3^Figure S14^.**. HRMS spectra of compound
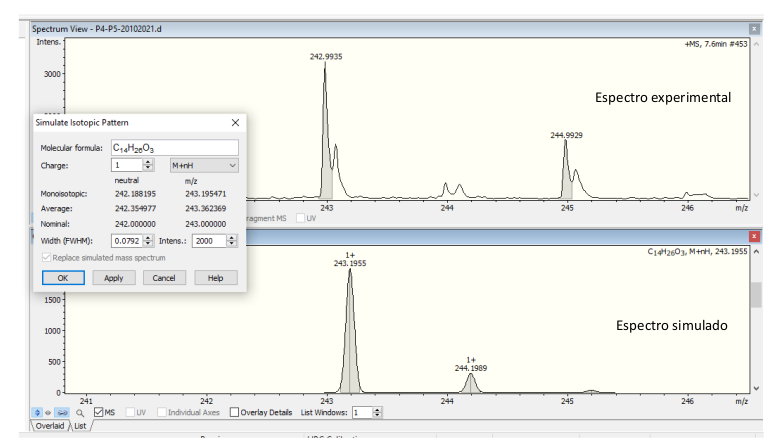
 **3**

**Figure S1**5. IR-FTIR spectra of
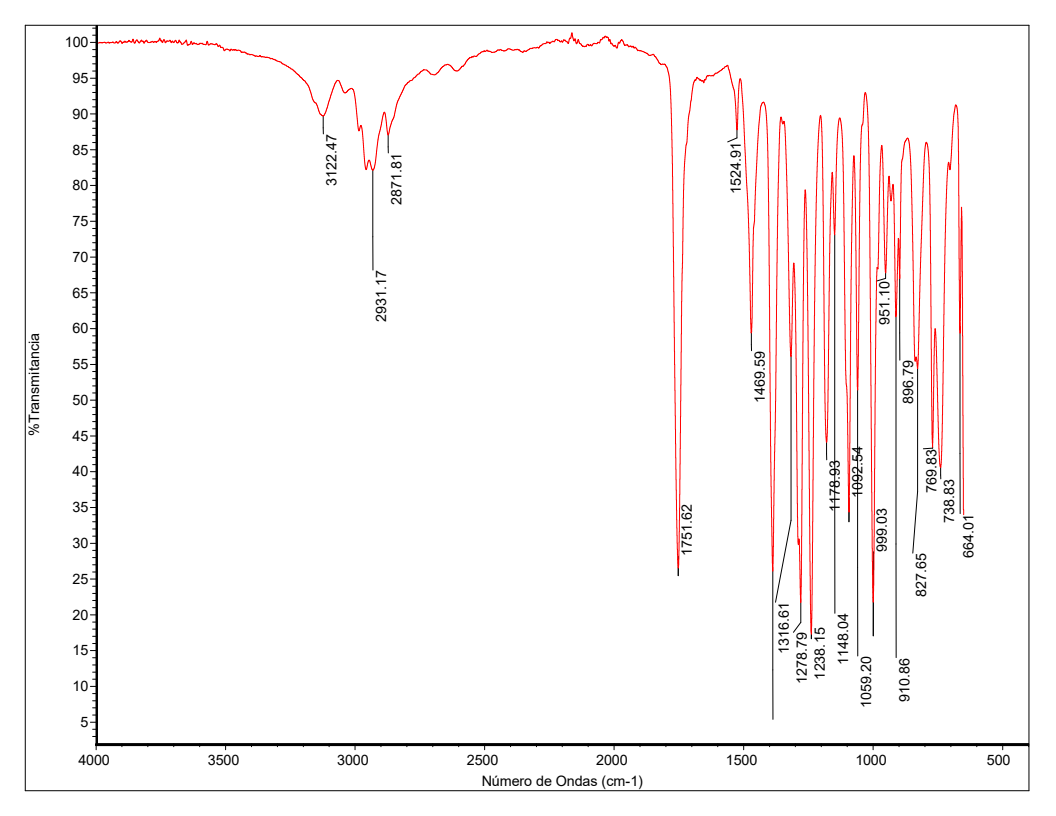
compound


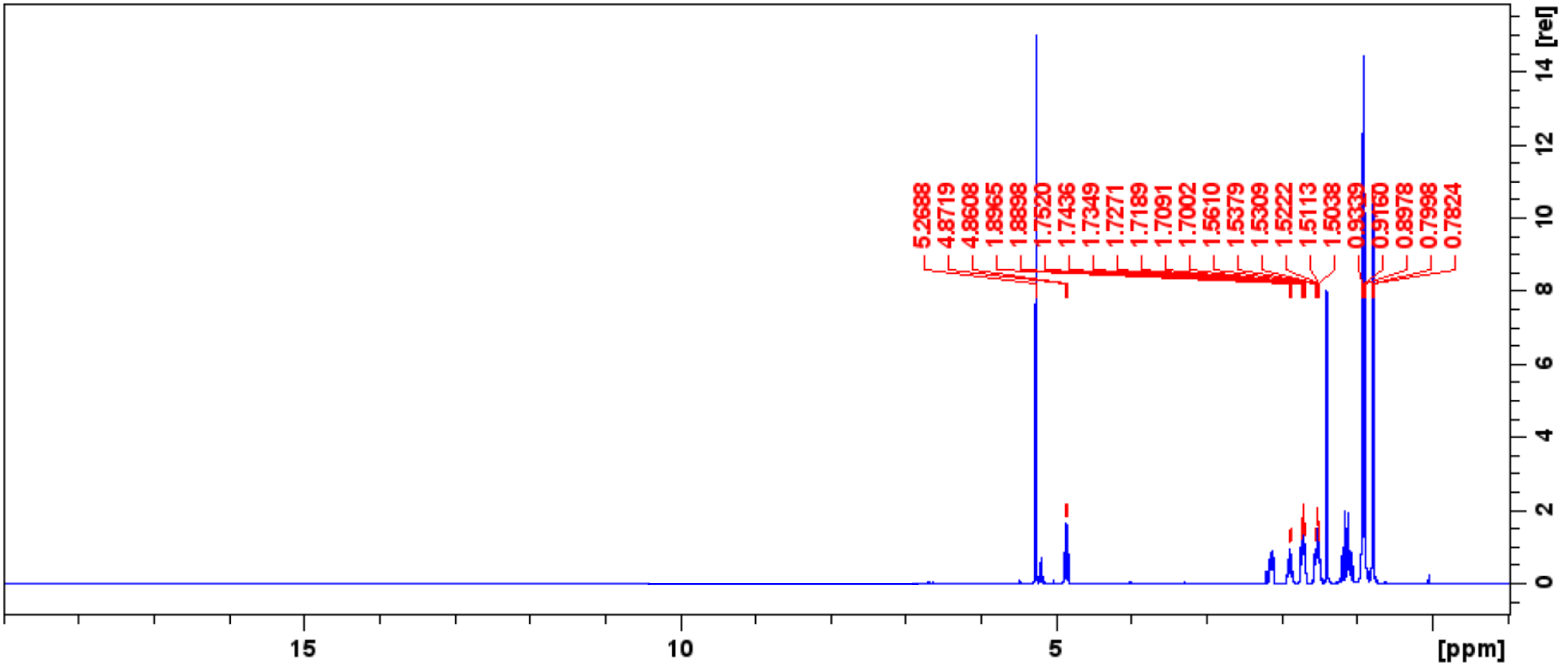
4

**Figure S16**. ^1^H NMR spectra of compound **4**

**
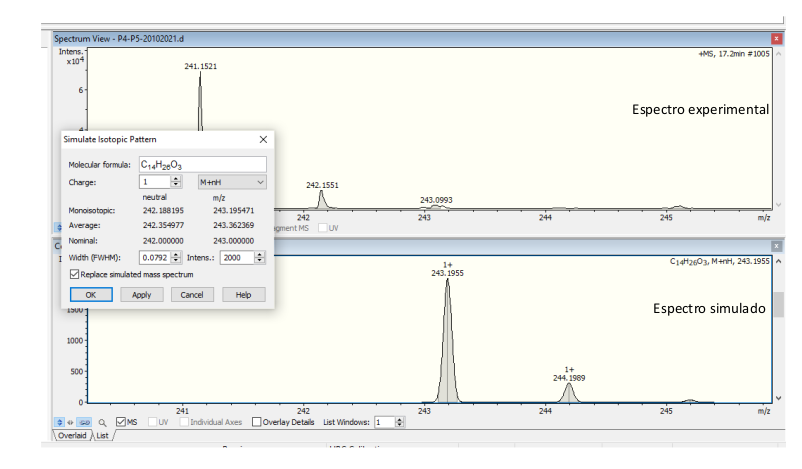
**

**Figure S17**. HRMS spectra of compound **4**

Figure S18. IR-FTIR spectra of compound
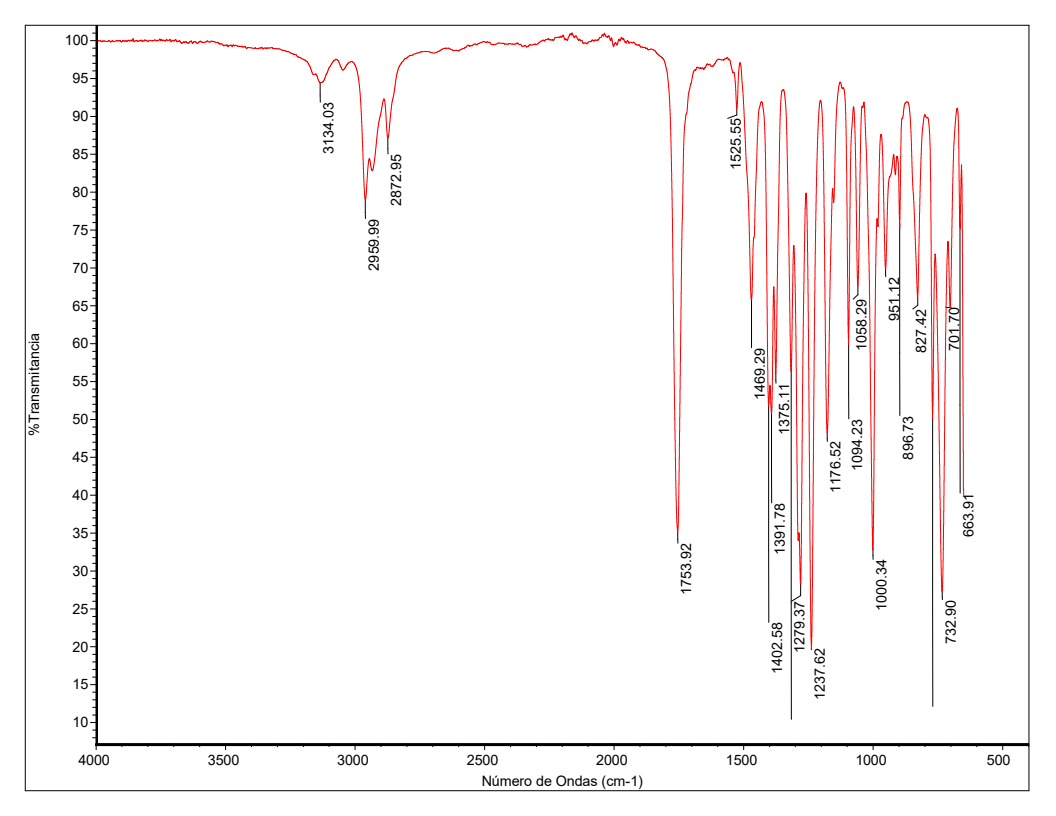
 **5**

Figure S19. 1H-RMN spectra of compound
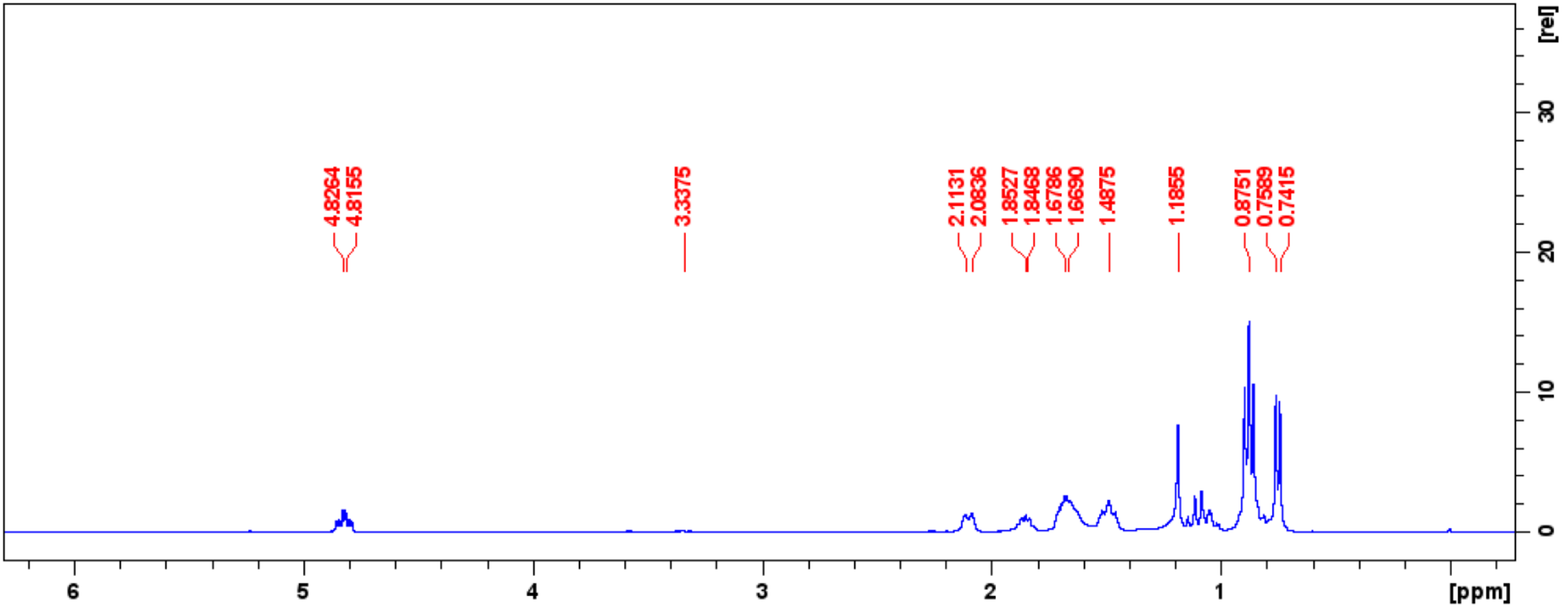
 **5**

**Figure S2**0. HRMS spectra of compound
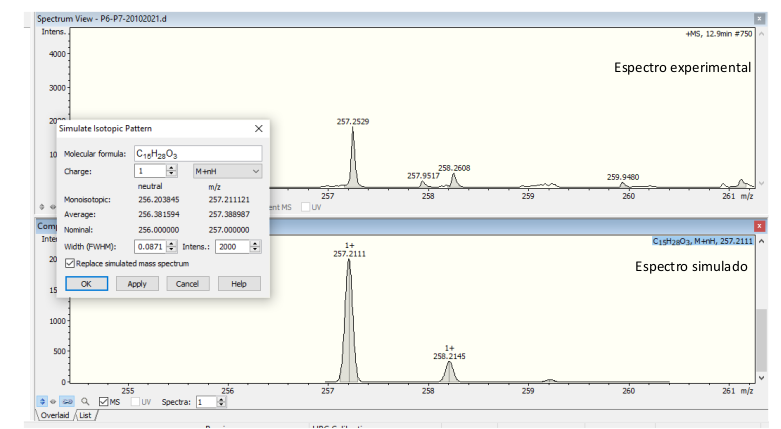
 **5**

5
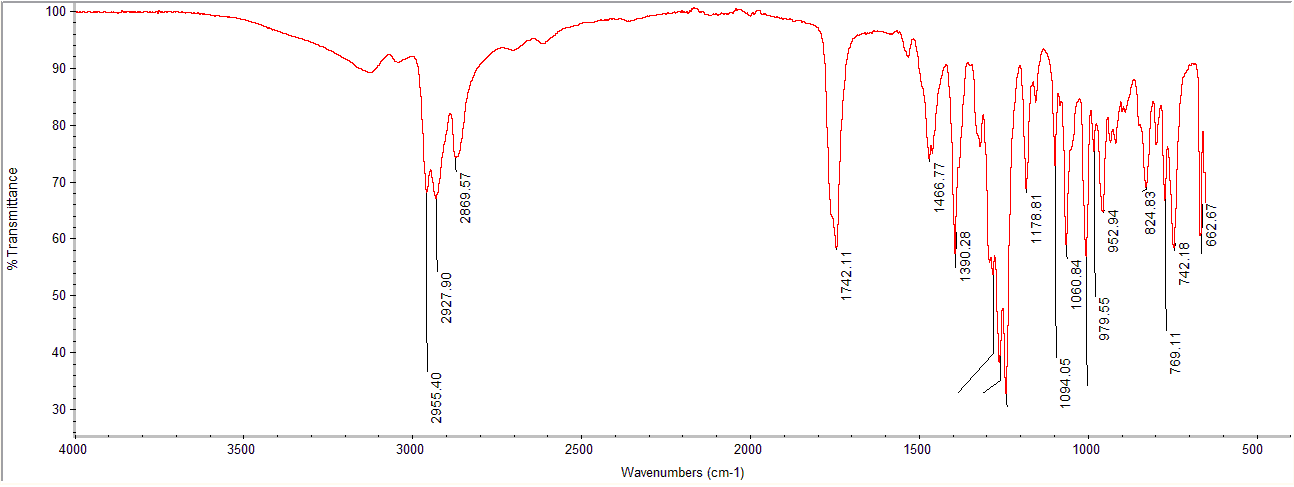


**Figure S21**. IR-FTIR spectra of compound **6**

**Figure S2**^2^. 1H-RMN spectra of
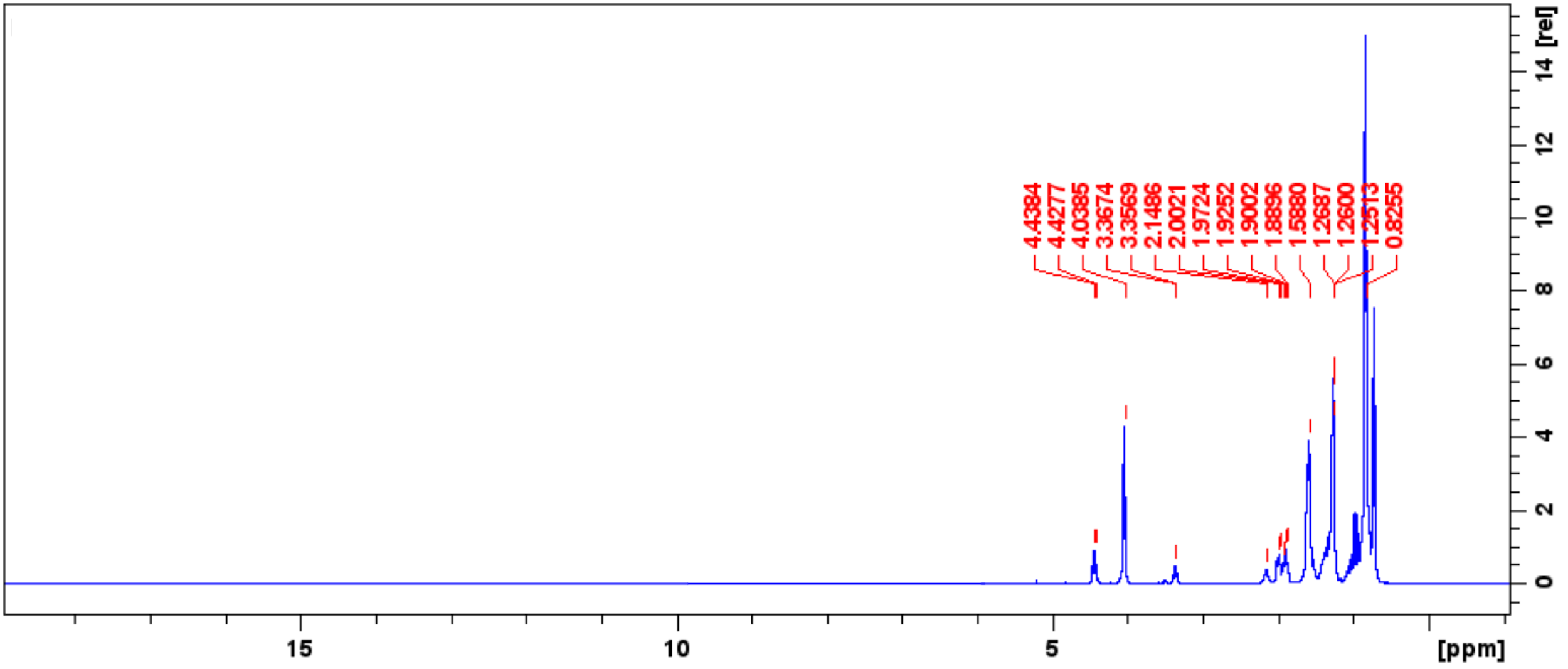
compound

**6**Figure S23. HRMS spectra of compound
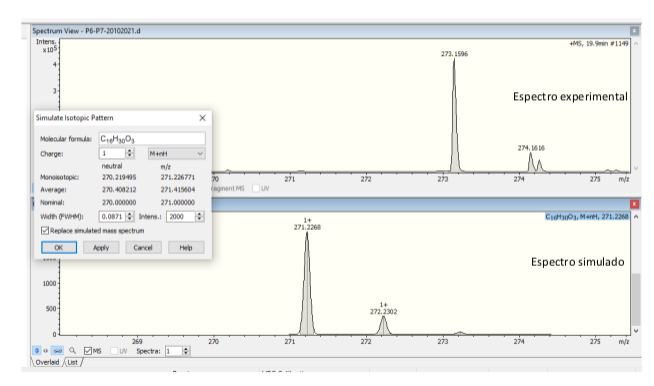
 **6**


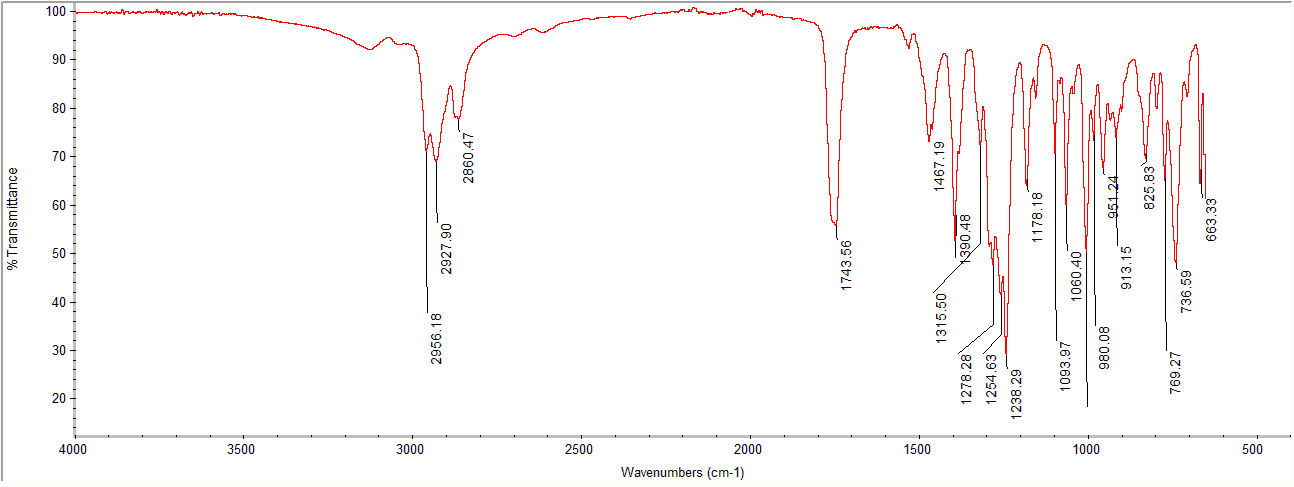


**Figure S24**. IR-FTIR spectra of compound **7**

**Figure S2**^5^. 1H-RMN spectra of compound
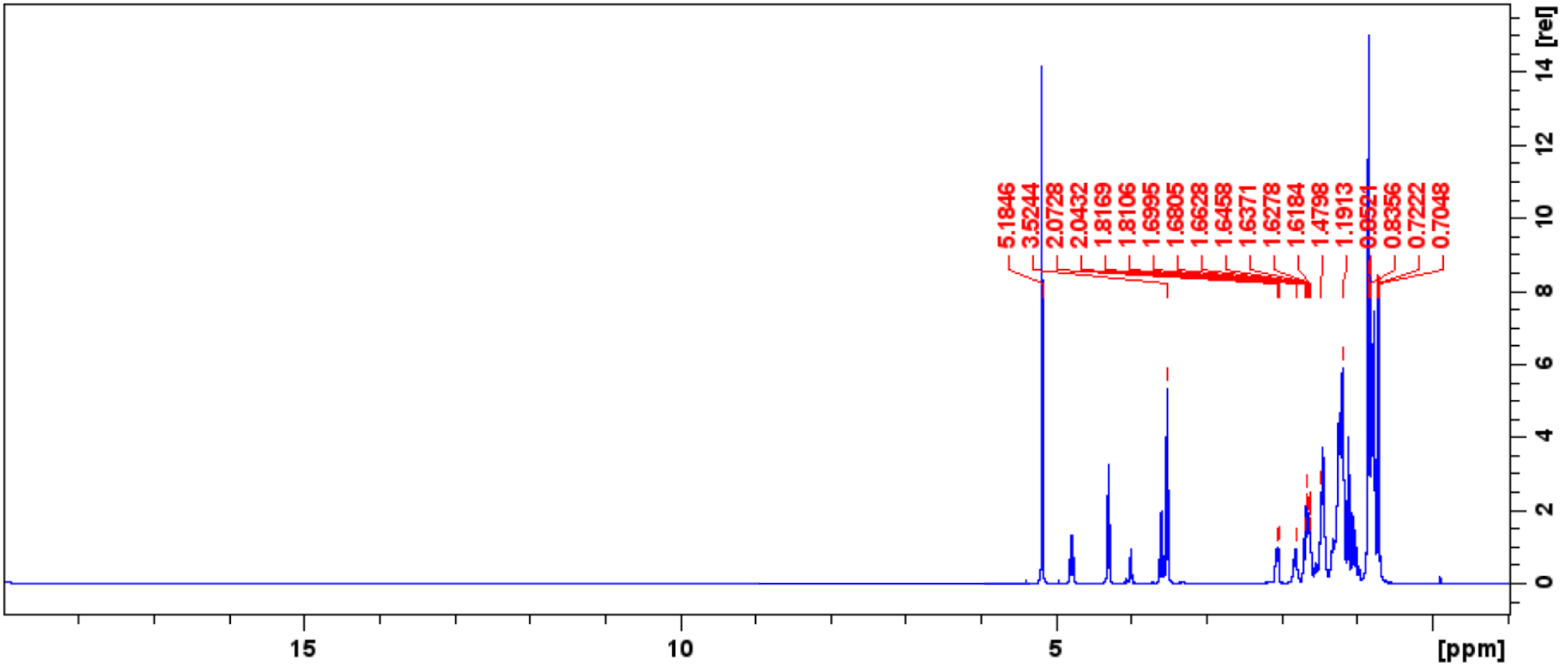
 **7**

Figure S26. HRMS spectra of compound
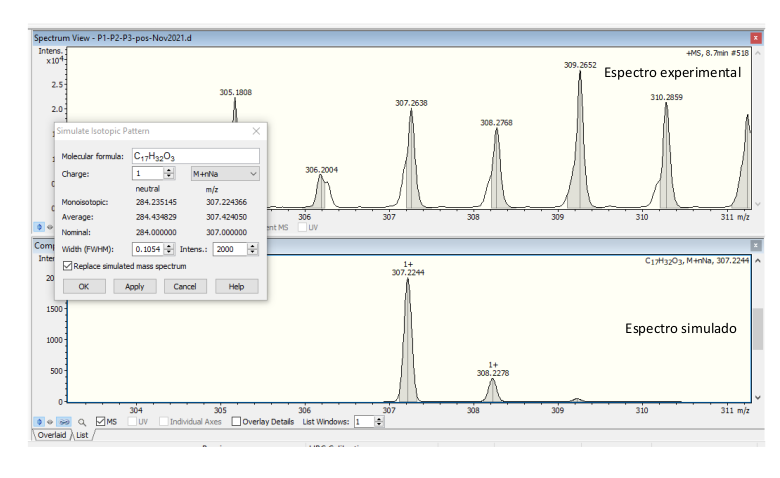
 **8**

7
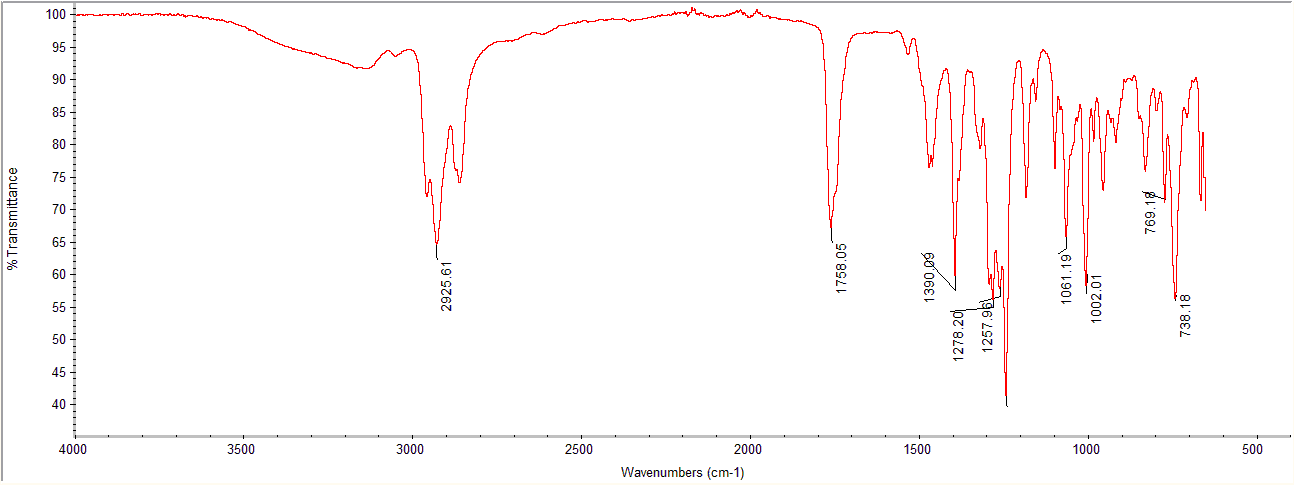


**Figure S27**. IR-FTIR spectra of compound **8**

**Figure S2**8. 1H-RMN spectra of compound
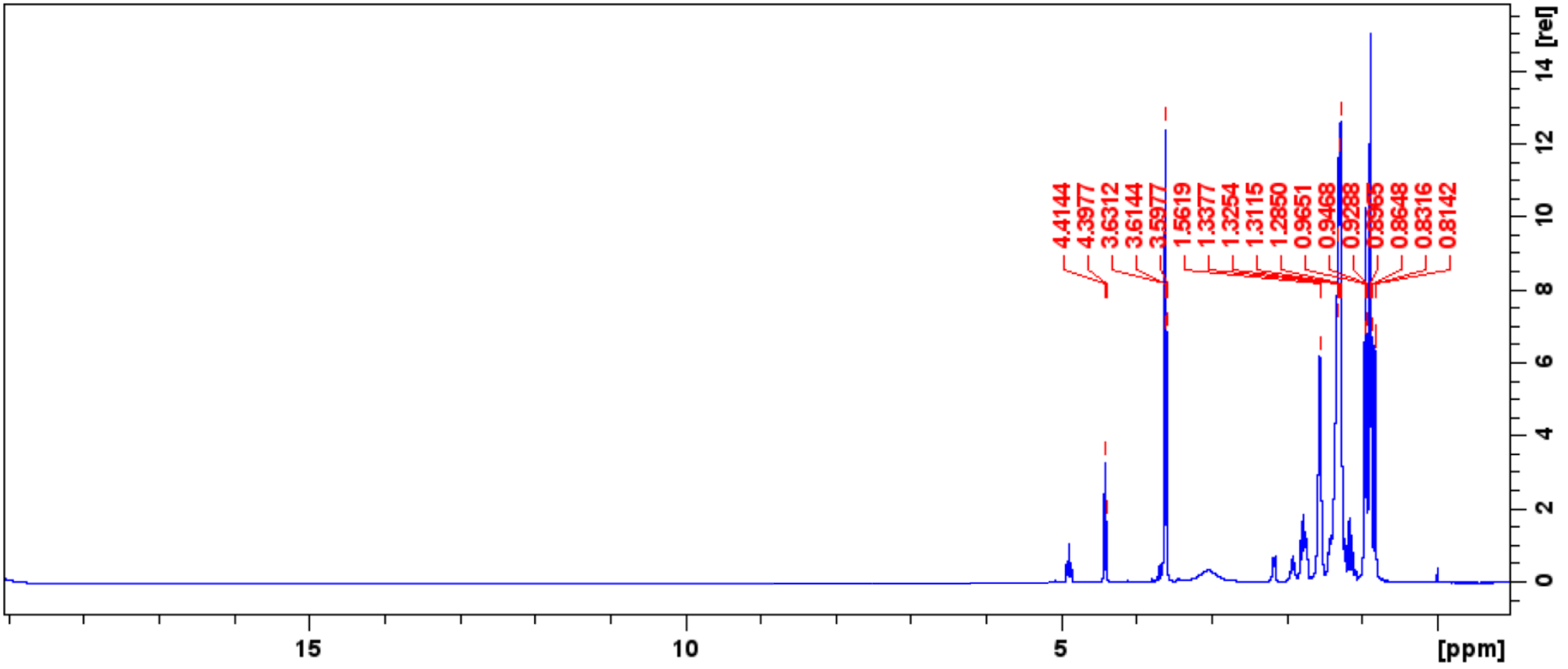
 **8**


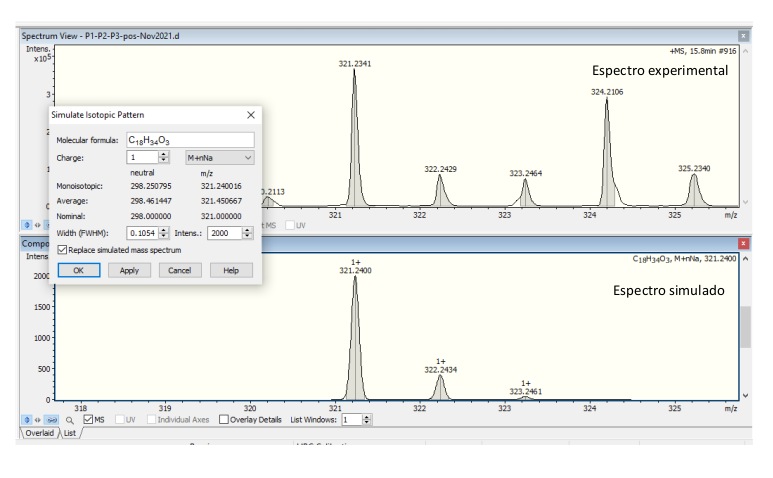


**Figure 29.** HRMS spectra of compound **8**


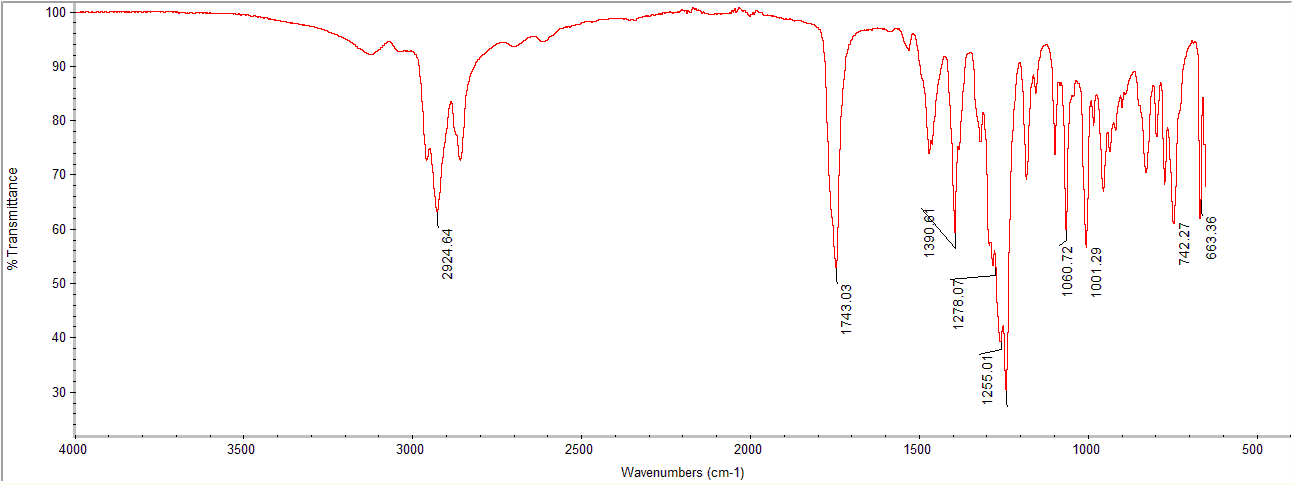


**Figure S30**. IR-FTIR spectra of compound **9**

**Figure S3**^1^. 1H-RMN spectra of
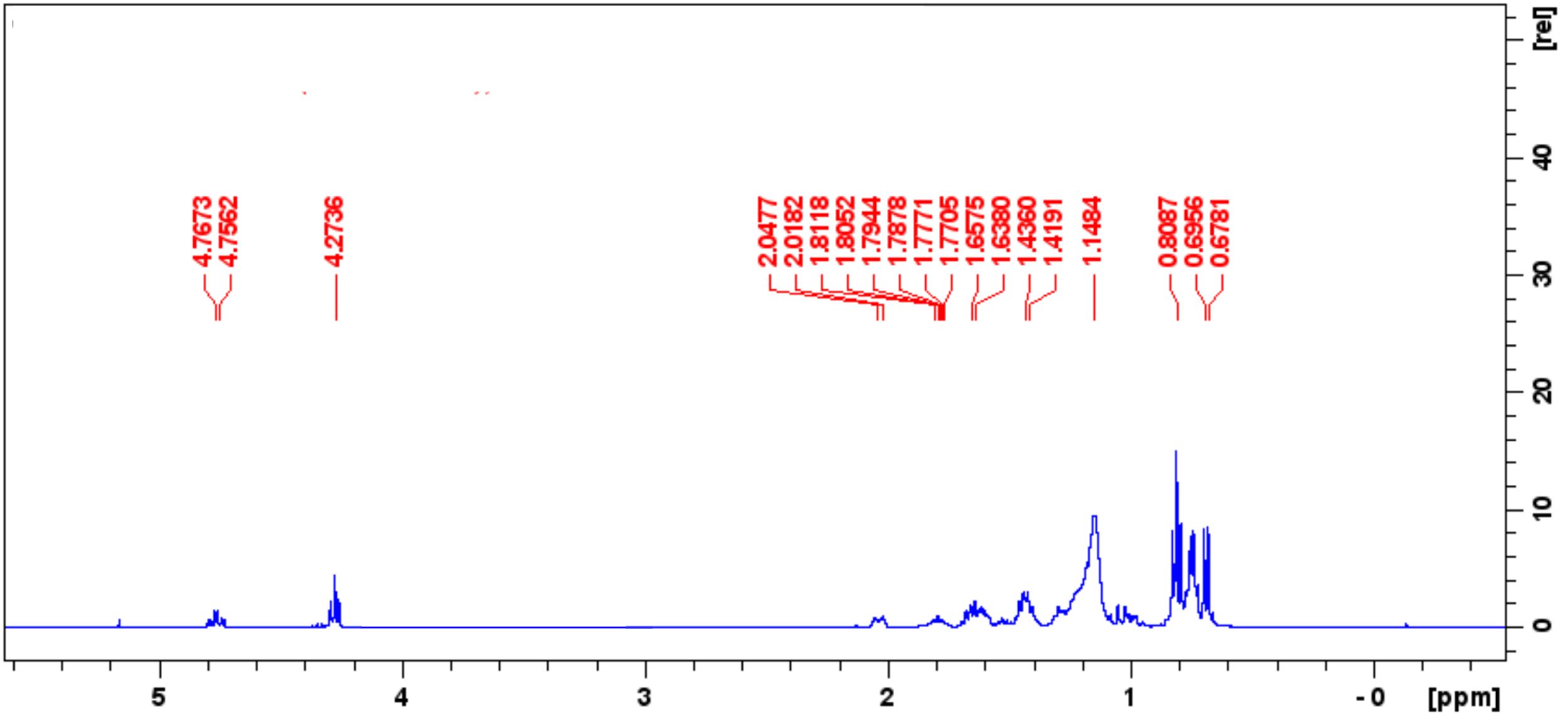
compound

9


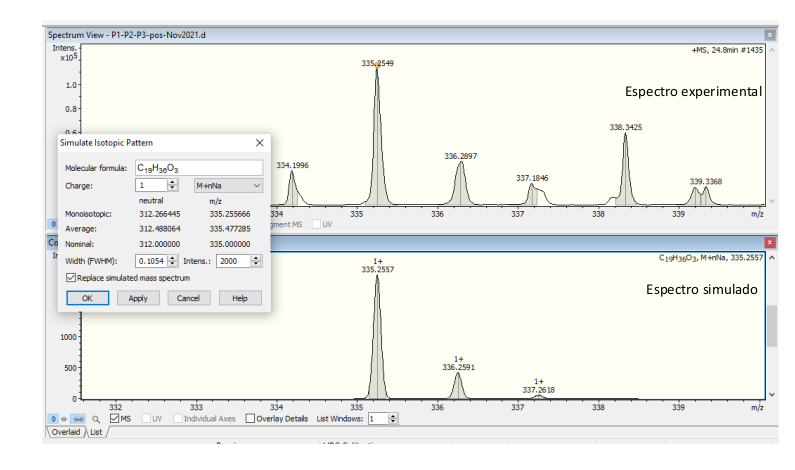


**Figure S32**. HRMS spectra of compound **9**
